# Structure Prediction of Complexes Controlling Beta- and Gamma-Herpesvirus Late Transcription Using AlphaFold 3

**DOI:** 10.3390/v17060779

**Published:** 2025-05-29

**Authors:** David H. Price

**Affiliations:** Department of Biochemistry and Molecular Biology, University of Iowa, Iowa City, IA 52240, USA; david-price@uiowa.edu

**Keywords:** herpesvirus, late transcription factors, AlphaFold 3, LTF complex, HCMV, MCMV, HHV6, HHV7, EBV, KSHV

## Abstract

All beta- and gamma-herpesviruses utilize a set of six viral proteins to facilitate transcription from specific promoters that become active late in the viral life cycle. These proteins form a complex that interacts with a TA-rich sequence upstream of the late transcription start sites and recruits RNA polymerase II (Pol II). The structure of any of the late transcription factors (LTFs) alone or in complexes has not been solved by standard means yet, but a fair amount is known about how the proteins interact and where the complex is positioned over the late promoters. Here, AlphaFold3 was used to predict and analyze the LTF complex using proteins from the beta-herpesviruses HCMV, MCMV, HHV6, and HHV7, and from the gamma-herpesviruses EBV and KSHV. The predicted structures had high levels of confidence and were remarkably similar even though there is little sequence conservation in the LTFs across the viruses. The results are consistent with most of the previously determined information concerning the interaction of the factors with each other and with DNA. A conserved threonine phosphorylation in one of the subunits that is critical to the function of the LTFs is predicted to be at the junction of five subunits. AlphaFold 3 predicts seven metal ion binding sites in each of the four beta-herpesviruses and either five or six in the gamma-herpesviruses created by conserved residues in three of the subunits. The structures also provide insights into the function of the subunits and which host general transcription factors (GTFs) may or may not be utilized during initiation.

## 1. Introduction

Herpesviruses are large double-stranded DNA viruses that infect cells from humans and other animals and carry out a complicated pattern of expression of hundreds of viral genes to combat the host anti-viral response, replicate the viral genome, and ultimately allow the virus to be packaged and spread to other cells [1,2]. All herpesviruses have genes that are transcribed by Pol II very early, early, and late, but beta- and gamma-herpesviruses have a set of six LTFs that are required for the expression of their late genes after viral DNA replication has started [3,4]. The very early and early genes are transcribed using the host-encoded GTFs, including TFIID (TBP with TAFs), TFIIA, TFIIB, TFIIE, TFIIF, and TFIIH, to form a preinitiation complex and help with early steps in Pol II initiation. One of the LTFs evidently substitutes for TBP because it interacts with a TATTA or TATAT sequence upstream of the late gene transcription start sites (TSSs) [5,6,7]. Unlike TBP, this LTF also interacts with Pol II through the C-terminal domain (CTD) of the large subunit [6,8]. It is not known if some of the other LTFs substitute for some or all of the host GTFs. What is clear from DFF-ChIP [9] experiments performed on HCMV-infected cells is that the LTF complex containing Pol II is very different from the normal TBP-driven transcription preinitiation complex (PIC) [10]. The TBP PIC protects 70 to 80 bp of DNA centered just upstream of the TSSs, while the LTFs protect only about 50 bp, all of which is upstream of the late TSSs [10]. While the LTF complex on late promoters is prevalent, it is relatively less efficient at driving transcription initiation compared to TBP PICs [10]. A large amount of work has provided incredible details about the structure and function of TBP PICs [11,12,13,14,15,16], but unfortunately, the structure of the LTF complex on a late promoter has not been solved despite efforts to do so [8].

Major advances in solving the structures of protein nucleic acid complexes have been made over the last two decades, especially through the use of cryo-EM [17,18], but many structures remain unsolved due to technical difficulties in producing the proteins and obtaining suitable images. Then, last year, came the Nobel Prize-winning work from Demis Hassabis, John Jumper, and David Baker that resulted in the successful utilization of artificial intelligence to predict protein structures and to design proteins from scratch. The AI algorithms developed by Google DeepMind, AlphaFold, have been demonstrated to accurately predict protein structures, and the most recent incarnation, AlphaFold 3, can predict structures of proteins complexed with nucleic acids and can include post-translational modifications of proteins as well as specific ligands (organic and ionic) [19]. The goal of the work presented here is to apply AlphaFold 3 to the LTFs to determine if a reasonable structure can be predicted and to use that information to help elucidate how the LTFs might function during initiation on late viral promoters.

## 2. Materials and Methods

### 2.1. Obtaining Representative Sequences for LTFs

Representative sequences of the six LTFs from HCMV, MCMV, HHV6, HHV7, EBV, and KSHV were obtained from the NCBI webserver. Because there are hundreds of sequences deposited for each, Identical Protein Groups was used to obtain the most highly represented sequence of each LTF. This should eliminate sequences that were obtained from mutated viruses or ones that were improperly truncated or extended. The collection of sequences used in this study is provided in Supplementary Data File S1. Because the nomenclature for the LTFs varies across the viruses, a common nomenclature was constructed using LTFa-f.

### 2.2. Multiple Sequence Alignment Using COBALT

The constraint-based multiple alignment tool (COBALT) from the NCBI webserver (https://www.ncbi.nlm.nih.gov/tools/cobalt/cobalt.cgi?CMD=Web) was used to compare homologous LTFs across the six viruses. This URL was utilized for this study many times between 1 January 2025 and 1 May 2025. When identical jobs were submitted at different times, nearly identical results were obtained. For the alignments, the View Format was set to “Compact” and the Conservation Setting was “Identity”.

### 2.3. Structure Predictions Using AlphaFold 3

The AlphaFold 3 server (https://alphafoldserver.com/) was used to obtain structure predictions. Hundreds of predictions were performed with a large variety of parameters for this study between 3 May 2024 and 1 May 2025. The general pipeline was to first input the desired protein sequences in the order LTFa to LTFf, make any post-translational modifications, and then, if applicable, add 60 bp of both strands of the strong late promoter DNA from the HCMV UL86 gene with LTFc recognition element (underlined) and transcription start site (bold): CGGCGTAGCGGAGCCGCCGACGACTATTAGAGCGTCACAGCCGAGGCGGCGCGGCGC**A**GC

For some jobs, a 30 bp truncated sequence was used:

GAGCCGCCGACGACTATTAGAGCGTCACAG

Finally, zinc ions were added when desired. Each job was saved with an informative name and submitted with the identical name, and once the job was completed, the 5 representative structures (.cif files) were downloaded and placed in an identically named folder. Initial analysis was performed in PyMOL (Version 2.5.5, Schrödinger, LLC, New York, NY, USA) by dragging the group of five structures into the program and using the Alignment plugin tool to orient the five structures. This last step made it easy to determine if the structures were highly similar or diverse. For the LTF complexes, all structures were highly similar. To view the AlphaFold 3 confidence, the AF3colors.py python script included as a Supplementary File was run to color the structure using the confidence values which are in the b factor column of the .cif files.

### 2.4. Calculation of Buried Surface Area

PyMOL was used to determine how much interaction there was between the DNA and proteins and between all of the subunits in the LTF complexes. After loading the desired LTF complex into PyMOL, the following three commands were run to allow the calculation of solvent (water)-accessible surface area: set dot_solvent, on; set dot_density, 4; set solvent_radius, 1.4. The surface area was determined using Action: compute, surface area, solvent accessible, for the entire complex. The DNA was then extracted as a separate object and then the surface area was determined for the DNA alone and the protein/zinc complex. The buried surface area was the difference between the total surface area and the sum of the surface areas of the DNA and protein/zinc complex. This was carried out for all six viral LTF complexes using the 30 bp DNA sequence and reported as a fraction of the total surface area. The buried surface area in the protein complex was calculated in a similar manner after determining the surface area of each individually extracted LTF with its associated zincs and comparing it to the surface area of the total protein complex.

## 3. Results and Discussion

**Figure 1:**
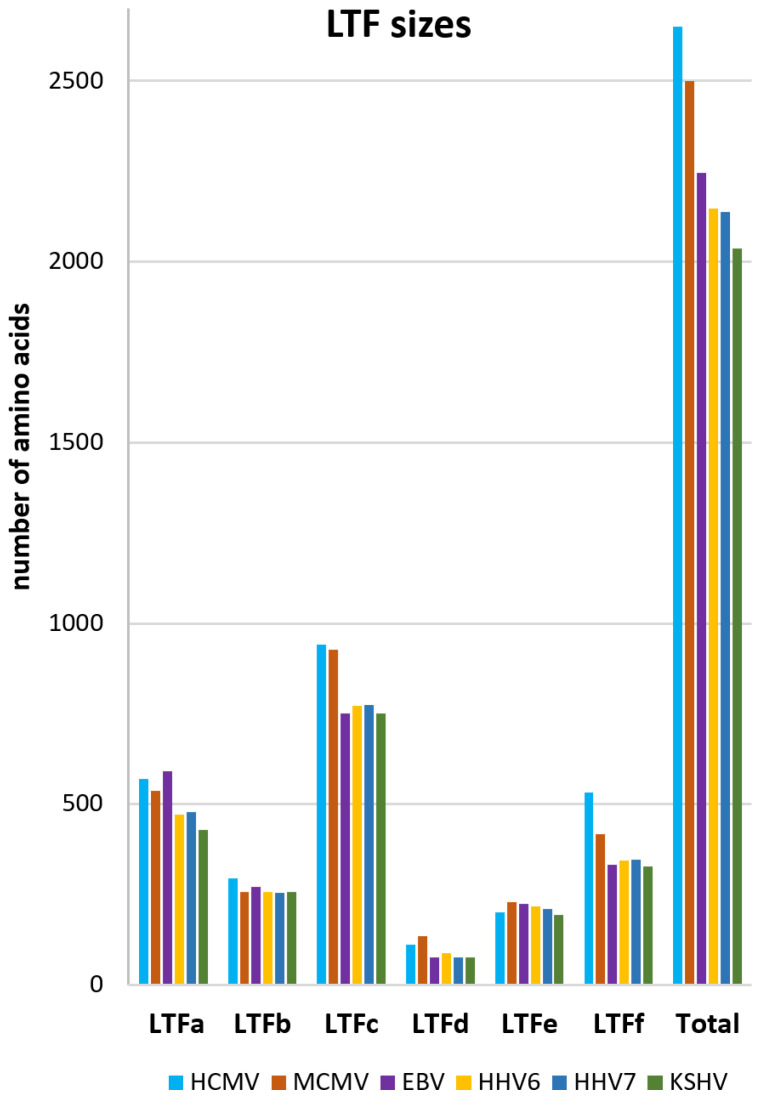
Sizes of LTFs and LTF complexes. The table and graph indicate the names and sizes (number of amino acids) of each of the LTFs from the six indicated viruses. How the individual sequences were chosen is described in Section 2.

AlphaFold 3 requires specific protein sequences to begin its analysis. Because there are a number of herpesviruses that utilize LTFs, the initial goal was to compile a list of LTFs from six different viruses that included four beta-herpesviruses, HCMV, MCMV, HHV6, and HHV7, and two gamma-herpesviruses, EBV and KSHV. The NCBI protein database has hundreds of sequences for each of the six LTFs in each virus, so the Identical Protein Groups database was used to pick the protein sequence with the largest number of entries which should correspond to the wildtype/prevalent sequence. Unfortunately, the nomenclature for the LTFs is non-uniform and a decision was made to create a common set of names, LTFa, LTFb, LTFc, LTFd, LTFe, and LTFf. Table 1 indicates how those general names correspond to each virus’s specific names and what the sizes of the proteins are. The sizes of each LTF and the size of the total for the six LTFs varied across the viruses, as shown in Figure 1. HCMV and MCMV had the most total amino acids (2651 and 2501, respectively) and had the largest LTFc’s. KSHV had the smallest LTFs and the least total amino acids (2036).

COBALT, the constraint-based multiple alignment tool served by NCBI, was used to analyze the similarities between each LTF across the six viruses. Supplementary Data File S1 has the sequences used in the analyses and the COBALT sequence comparison for each LTF. The numbers of identical amino acids across the six viruses for each LTF were relatively small (LTFa, 35; LTFb, 26; LTFc, 83; LTFd, 2; LTFe, 17; LTFf, 26). Overall, the 189 identities were less than 10% of the total number of amino acids. As expected, the C-terminal domain of LTFc, which is known to interact with the upstream TA-rich sequence found in late promoters, had the highest percent identity. That domain has homology with TBP [5], but there were only 10 identical amino acids when COBALT analysis was performed on the six LTFc’s and TBP together (see Supplementary Data File S1).

**Figure 2:**
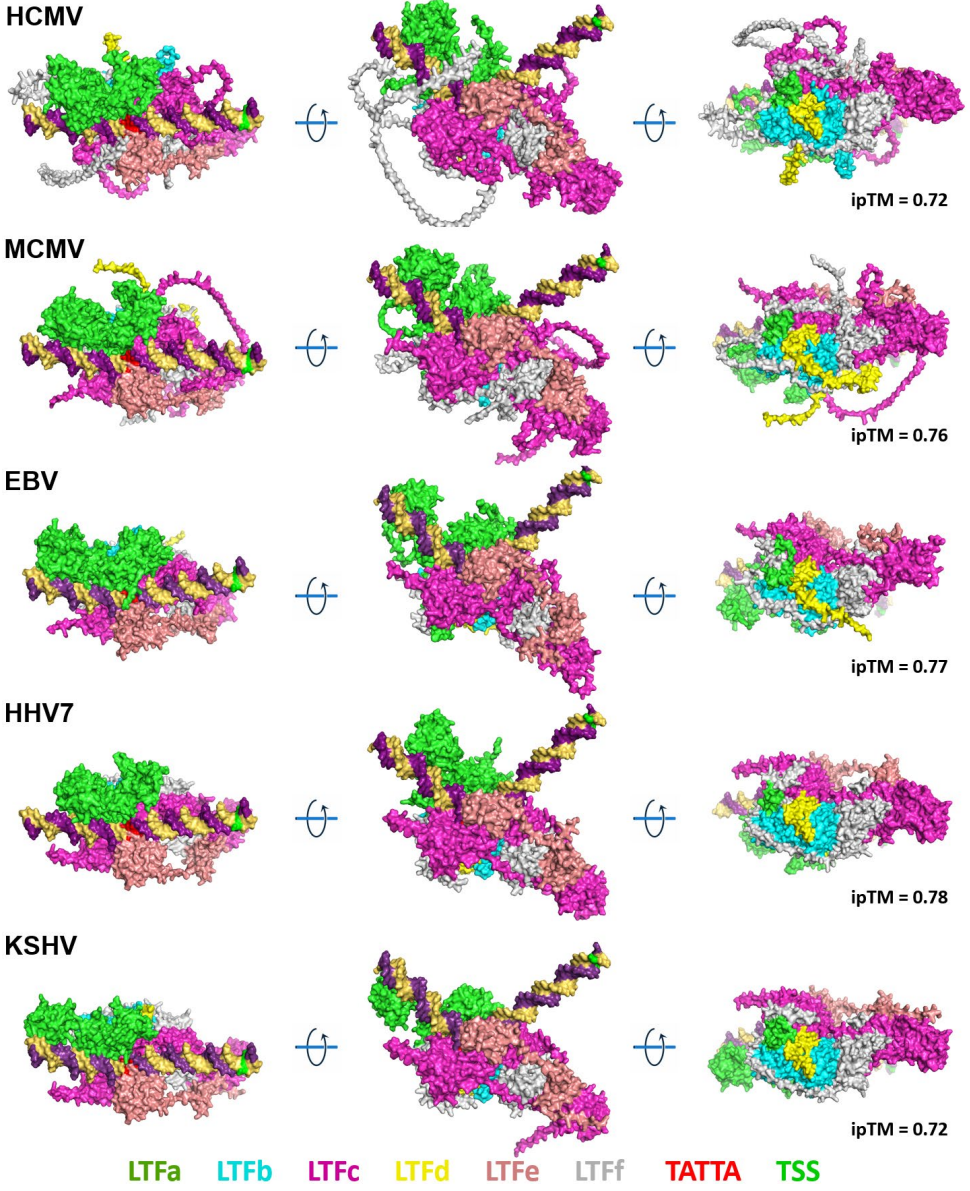
AlphaFold 3-predicted structures of the LTF complex on viral late promoter DNA. The complete amino acid sequence of each LTF, the conserved LTFf threonine phosphorylation, and zinc ions were included in each job. The three views for each complex are 90° rotations. Supplemental Data File S2 is a PyMOL session with HCMV, MCMV, EBV, HHV6, HHV7, and KSHV structures aligned.

Hundreds of AlphaFold 3 jobs were created and submitted before settling on the appropriate parameters for generating the highest-confidence structures. Individual LTFs and groups of LTFs were tried. For each virus, the structures of the six LTF complexes with and without a 60 bp DNA sequence from the late promoter driving HCMV UL86 were submitted. The initial results indicated that similar structures were generated from each virus with or without DNA. In addition, a threonine phosphorylation in EBV BGLF3 (LTFf) that has been demonstrated to be required for efficient LTF function [20] and seven Zn ions for the beta-herpesviruses, five Zn ions for KSHV, and six for EBV were added to the jobs submitted. The structures generated using these parameters for the six LTFs bound to the HCMV UL86 late promoter for the six viruses had a highly conserved arrangement of the LTFs (Figure 2). Note that HHV6 is not shown because of its very high similarity to HHV7. The ipTM values generated during the structural predictions were between 0.72 and 0.78, which suggests that the predicted structures may have significant accuracy. The HCMV and MCMV complexes had the largest fraction of unstructured regions (loops), and these corresponded with insertions in the protein sequences found in the COBALT analyses. For example, the first 96 residues of HCMV LTFf and the first 28 residues of MCMV LTFf were identified as insertions and were unstructured. Almost no unstructured regions were found in the HHV6, HHV7, EBV, and KSHV LTF/promoter complexes. Of critical importance is the interaction between the LTF complexes and the promoter DNA. The region of LTFc with structural homology to TBP was directly associated with the TATTA sequence in the promoter (colored red), and the DNA was bent at about 120 degrees at that site. This is similar to how TBP interacts with and bends its recognition sequence [21]. It is likely that the predicted conformation of the DNA was derived from the conservation between LTFc and TBP [5] coupled with the known structure of TBP bound to its TA-rich recognition sequence, leading to a dramatic bend in the DNA [22,23]. Importantly, the AlphaFold 3-predicted structures are completely consistent with the size and positioning of the LTF complex upstream of the transcription start site as determined by DFF-ChIP in HCMV-infected primary human foreskin fibroblasts [10].

**Figure 3:**
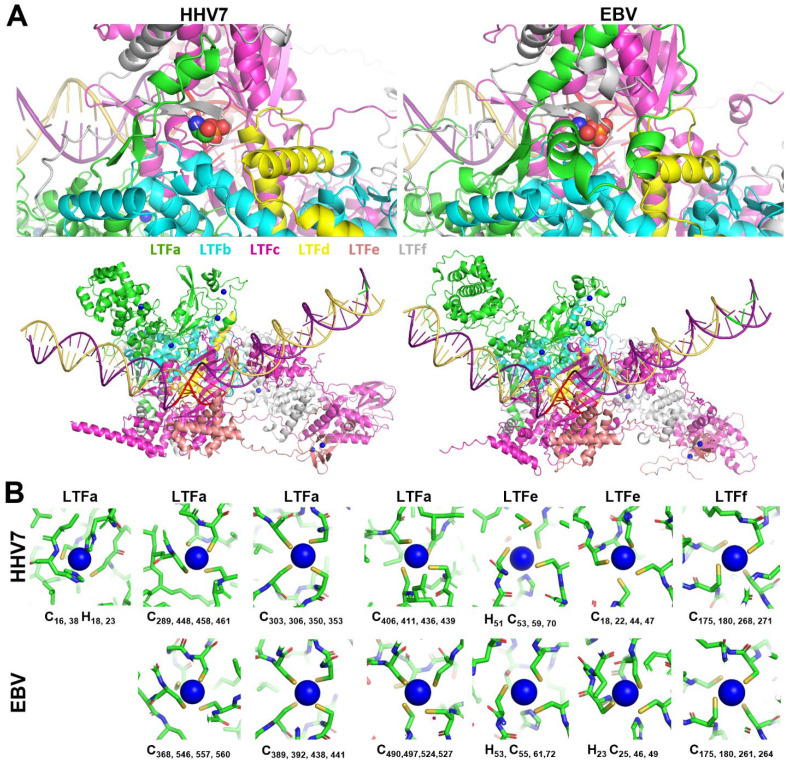
LTFf phosphorylation and metal binding in LTF complexes. (**A**) Phosphorylation of the conserved threonine in LTFf (T50 in HHV7 and T42 in EBV) shown as colored spheres (**top**) and the location of the zinc ions in HHV7 and EBV (**bottom**). (**B**) Detail of the zinc binding sites in the indicated LTFs showing the ionic interacting residues. The residues involved in zinc binding for all viruses are highlighted in the sequence comparisons in Supplementary Data File S1.

All LTFf’s contain a conserved threonine, and phosphorylation of that residue has been shown to be essential for EBV LTF function [20]. The phosphorylated threonine was predicted to be in the middle of a conserved N-terminal domain of LTFf, and the position of the phosphate in the LTF/promoter structure is at the junction of five LTFs, as illustrated by HHV7 and EBV (Figure 3A, top). The phosphate is found in a small, deep pocket at the interface of LTFa, b, c, d, and f in all six of the viral LTF complexes. The pocket is present even in the LTF/promoter structures generated without the phosphorylation. Neutralization of the negative charge of the phosphate is accomplished in part by interactions of several backbone nitrogens and perhaps water molecules that could be accommodated in the deep pocket. The inclusion of threonine phosphorylation in the AlphaFold 3 predictions led to an increase in the confidence values for many residues across the interfaces between the EBV subunits (Supplementary Figure S1).

**Figure 4:**
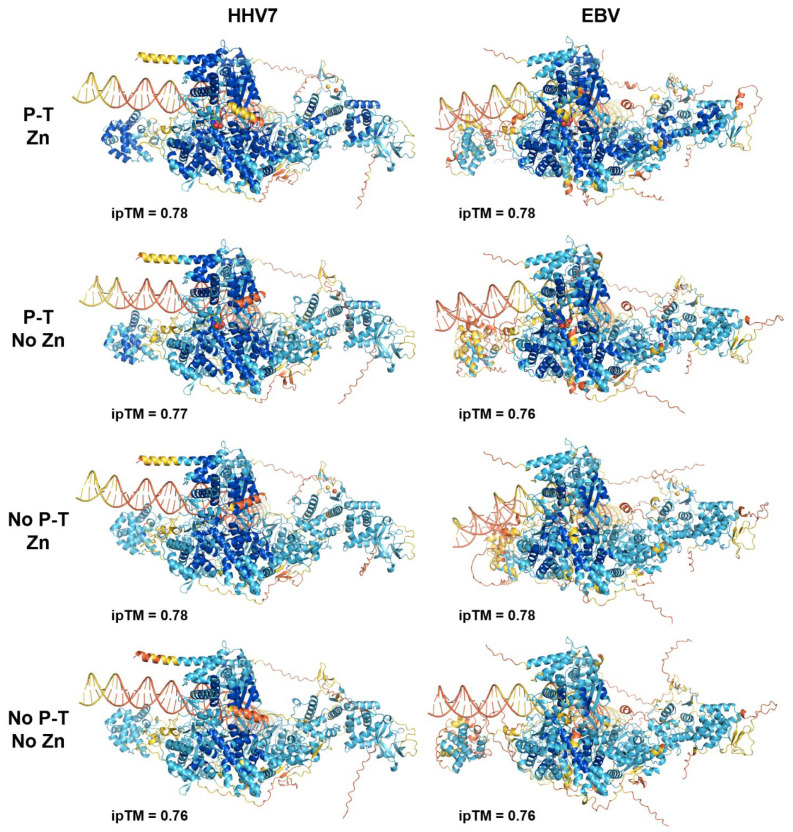
AlphaFold 3 prediction confidence without and with LTFf phosphorylation and zinc ions. The indicated viral LTF complexes have been colored using the AlphaFold 3 confidence values. P-T, phosphothreonine in LTFf.

Evidence is mounting for metal binding playing a role in the function of the LTFs. Two different groups have demonstrated that conserved Cx_n_C motifs found in LTFa [24] and in LTFf [25] are important for the function of KSHV LTFs. Because of this, zinc ions were titrated into AlphaFold 3 jobs with all six sets of six LTFs (including LTFf with threonine phosphorylation) with a late promoter. For the two gamma-herpesviruses, there were five (KSHV) or six (EBV) specific sites occupied by zinc. However, for the four beta-herpesviruses, there were seven specific sites of occupancy. The location of the zincs is illustrated by the HHV7 and EBV LTF complexes (Figure 3A, bottom). At least two zincs were found in LTFa in all viruses, with EBV LTFa containing three and all beta-herpesvirus LTFa’s containing four. Two were found in LTFe and one in LTFf in all viruses (Figure 3A, bottom). In general, each zinc was chelated by groups of four conserved cysteines and or histidines (Figure 3B). The lower numbers of zincs in EBV and KSHV were accompanied by the loss of conserved residues in LTFa in those viruses (see sequence comparisons in Supplemental Data File S1). Another difference between virus types was that one of the zincs in LTFe from KSHV had only two cysteines. Because of this, it is not clear if zinc actually binds to that site. Overall, the phosphorylation of LTFf and the addition of zincs improved the confidence of the prediction of all of the viral LTF complexes, and this correlated with increasing ipTMs for most viruses when the phosphothreonine and zincs were added (Figure 4). Combined with the earlier experimental data, it seems likely that zinc binding plays a major role in the structure and function of the LTF complex.

**Figure 5:**
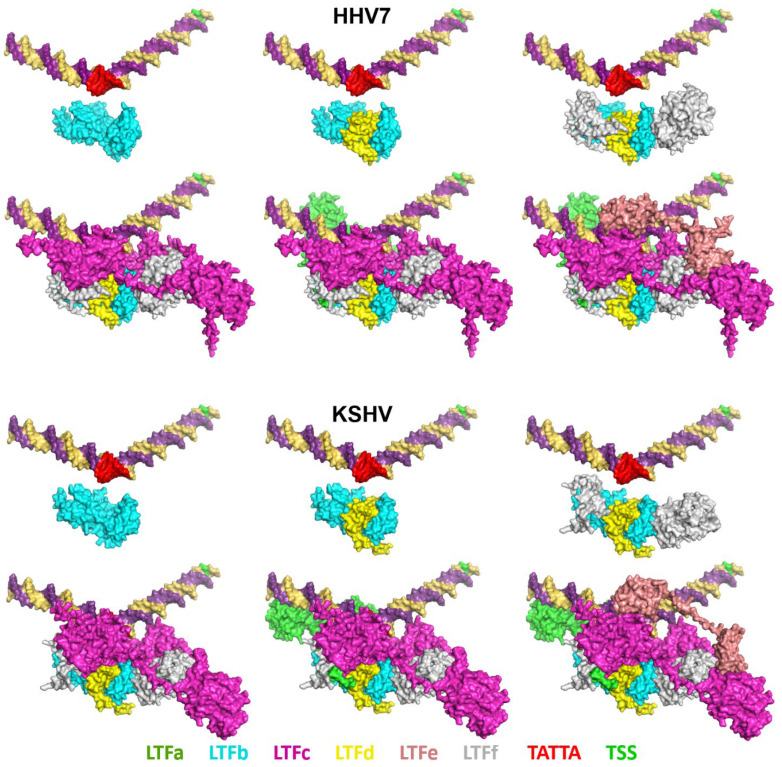
LTF interactions with each other and with promoter DNA. Individual LTFs were extracted from the HHV7 (top) and KSHV (bottom) LTF complexes with LTFf threonine phosphorylation and zincs and shown as surfaces. The individual LTFs are color-coded as indicated, as are the TATTA sequence and the transcription start site (TSS) in the promoter DNA. Supplemental Data File S3 is a PyMOL session of the HHV7 LTF complex with extracted subunits.

The structures of the LTF complexes have a mix of LTF interactions with each other and with the promoter DNA. The six viral LTF complexes all share the same interactions. For illustrative purposes, the KSHV and HHV7 complexes are compared in Figure 5. LTFb and LTFd have a major interaction with each other, as has been shown [26], and LTFf interacts with the LTFbd complex. None of those subunits have any direct interaction with the promoter DNA. LTFc bridges the LTFbdf complex to the promoter through the interaction of LTFc with the upstream TATT element. LTFc extends in a downstream direction with minimal DNA contacts, but with a major interaction with LTFf. LTFa interacts with the DNA mainly upstream of TATTA and interacts with LTFc and LTFb. LTFe binds to the DNA, and each of its two domains binds only to LTFc. The C-terminal domain of LTFe binds near TATTA and interacts with the TBP-like domain of LTFc. The N-terminal domain associates with the downstream lobe of LTFc that has been implicated in binding to the CTD of the large subunit of Pol II [6,8]. To quantify the extent of protein/protein interactions, the buried surface area was calculated for the proteins in all viral LTF complexes. The percentage of the protein surface area that was buried was quite high at 20% to 28% (Supplementary Figure S2). Not surprisingly, HCMV and MCMV had the lowest values due to the inability of the unstructured regions specific for those viral complexes to interact with other regions of the complex.

**Figure 6:**
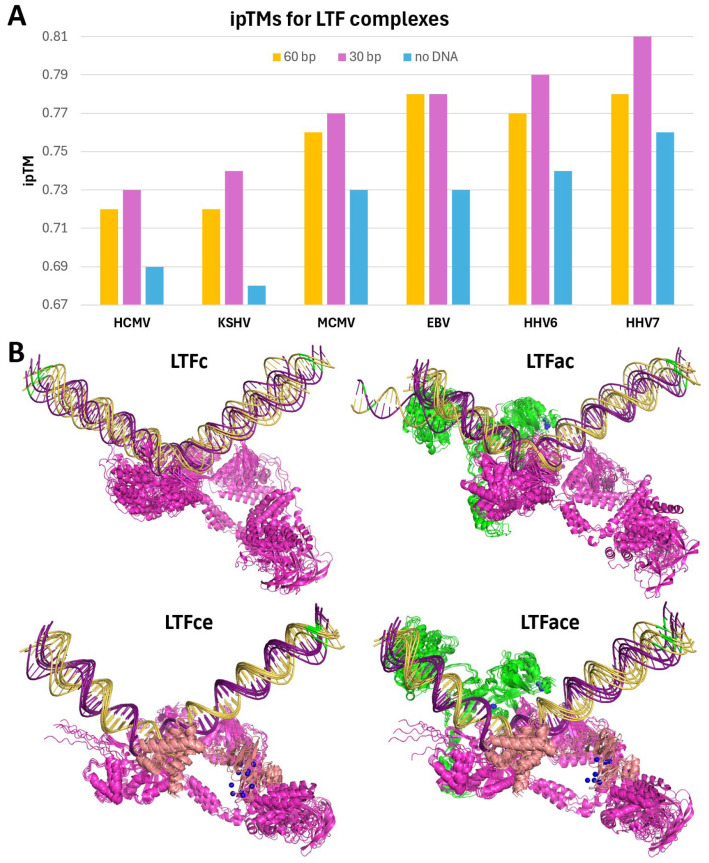
Involvement of DNA in LTF complex structural predictions. (**A**) The graph indicates the ipTMs for structures predicted for the six LTF complexes containing LTFf phosphothreonine and five zincs for KSHV, six for EBV, and seven for the rest with either 60 or 30 bp of DNA or no DNA. (**B**) The six predicted structures for the indicated KSHV complexes were aligned. The position of the TSS in the DNA is indicated by the green base pair.

To quantify how DNA might affect the prediction of the LTF complexes, jobs with different lengths of DNA or with no DNA were run while maintaining the LTFf phosphorylation and zinc ions for all six viruses. Compared to the 60 bp promoter, a 30 bp DNA centered on the TA-rich recognition element in the middle improved the ipTM scores of all viral LTF complexes (Figure 6A). This was somewhat expected since much of the 60 bp DNA was of low confidence and reducing the amount of it would increase the fraction of the atoms with higher confidence. However, when the DNA was removed from the jobs, the ipTMs fell significantly. The percentage of the surface area buried by DNA was between 3% and 5%, again with HCMV and MCMV having the two lowest values due to the presence of unstructured protein segments (Supplementary Figure S2). Together, these results indicate that DNA plays a significant role in stabilizing the LTF complexes.

To examine the interdependence of the three LTFs that bind to DNA, AlphaFold 3 was used to predict the structures of LTFa, LTFc, and LTFe from KSHV (Supplementary Figure S3) and HHV7 (Supplementary Figure S4) with and without promoter DNA and with and without Zn for LTFa and LTFe. LTFa folded into a three-lobe structure, and the addition of Zn improved the confidence of the Zn binding domain. Interestingly, LTFa only bound to late promoter DNA when Zn was included; however, it did not bind in the proper location seen in the full complex. The Zn binding domain of LTFe also folded into a more confident structure in the presence of Zn, but the two domains of LTFe that occupy very different locations in the complete LTF structure were right next to each other. Like LTFa, LTFe was predicted to bind to DNA in the presence of Zn and did not bind in the correct position. AlphaFold 3 predicted that KSHV LTFc alone would bind to the TATTA sequence in the late promoter, but of the five structures generated, only two were bound in the correct orientation seen in the complete LTF structure (Figure 6B). The addition of LTFa to LTFc improved the directionality of LTFc (four of six structures were in the correct orientation). The addition of LTFe to LTFc led to all six structures being in the proper direction and caused the two domains of LTFe to separate as they are found in the full LTF complex. The LTFace complex maintained the proper positions of the three factors and the correct orientation. Overall, the conclusion drawn is that LTFc is the primary DNA binding factor and that LTFa and especially LTFe are required to provide proper directionality to the interaction with DNA.

**Figure 7:**
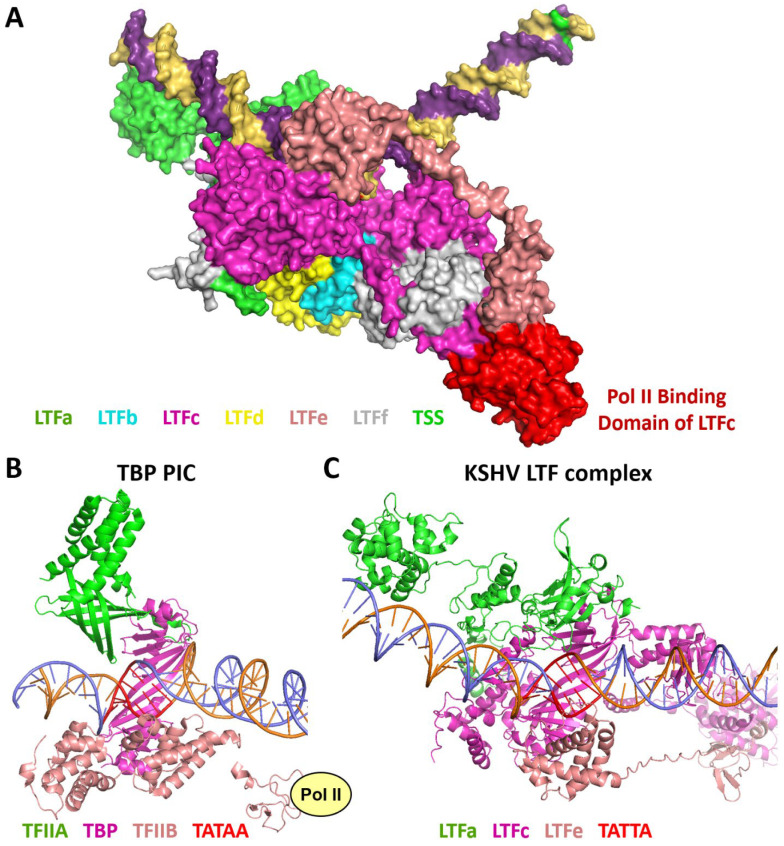
Functional aspects of the LTF complex structure. (**A**) KSHV LTF complex with the Pol II binding domain of LTFc in red. (**B**) TBP PIC from PDB 5IY6 [21]. TATA promoter with TBP, TFIIA, and TFIIB. The domain of TFIIB that interacts with Pol II is indicated. (**C**) KSHV LTF complex with the same orientation as the TBP PIC.

There is a vast amount of information about the structures of TBP-containing PICs and the function of the individual GTFs [11,12,13,14,15,16], but how the LTF complex compares to that is mostly unknown. The major difference is that the TBP PIC covers more DNA that includes the transcription start site and downstream sequences [11,13,14,15,16,21], while the LTF complex only covers DNA upstream of the TSS [10]. This means that Pol II, which must interact with the TSS, is not in position to initiate transcription in the LTF complex. The domain of LTFc that has been shown to interact with Pol II [8] is the domain that is farthest downstream (Figure 7A), and its interaction with the CTD of Pol II could help Pol II locate the TSS; however, AlphaFold 3 failed to predict a specific interaction of the CTD with or without phosphorylation with this domain of LTFc. In the TBP PIC, TFIIB is an essential GTF that has two separate domains [11,12,21]. One interacts with upstream DNA and TBP bound to its recognition sequence and the other binds directly to Pol II (Figure 7B). TFIIA is also involved by binding to upstream DNA and TBP (Figure 7B). Note that if looking down at the template toward the TSS with TBP on the bottom, TFIIA is on the left side of the DNA and TFIIB is on the right side. By aligning TBP and the TBP-like domain of LTFc, LTFa is in the same position as TFIIA and LTFe is in the same position as TFIIB (Figure 7C). This suggests that LTFa and LTFe substitute for the two GTFs. Interestingly, both TFIIB and LTFe each have two tethered domains. One interacts with TBP or the TBP-like domain in LTFc and the other interacts with Pol II directly or with the Pol II binding domain of LTFc. Clearly, LTFe would block the entry of TFIIB and LTFa would block the entry of TFIIA, and this was borne out by the failure of AlphaFold 3 to incorporate TFIIB or TFIIA into discrete positions in the complete LTF complex. When TFIIB was used in place of LTFe to generate an LTF complex, TFIIB was positioned exactly as it is in the TBP PIC, with TFIIB interacting with the TBP-like domain of LTFc and the right-hand side of the DNA. It is not clear if TFIIB is required for LTF-driven initiation, but if it is, it would require the removal of LTFe.

## 4. Conclusions

AlphaFold 3 was able to predict a complicated structure of six LTFs from six different viruses bound to DNA containing a late promoter with significant confidence. There was a high level of structural similarity between all six viruses. Importantly, the confidences of the structures were positively influenced by the inclusion of Zn and threonine phosphorylation of LTFf. LTFb, LTFd, and LTFf interacted extensively with each other, but not the DNA, and LTFa, LTFc, and LTFe interacted with the LTFbdf complex and DNA. LTFc interaction with the TATTA late promoter motif was the driving force for promoter occupancy, with LTFa and LTFe improving the directionality of the complex. It is interesting that LTFa was located in a similar position to TFIIA in the TBP PIC and that LTFe was positioned like TFIIB. It is possible that there is no requirement for TFIIA and TFIIB for LTF-driven transcription. Deciphering this will require experimental evidence which will be complicated by the fact that most late promoters have a mixture of TBP PICs and LTF complexes [10]. The structures presented here should be useful in predicting interactions between the LTFs that could be tested using biological experiments and in helping to solve cryo-EM structures. Since DFF-ChIP experiments gave similar positioning and DNA protection [10] to the predicted structures, DFF-ChIP may be an excellent starting point for the isolation of native LTF complexes for cryo-EM. 

## Figures and Tables

**Table 1:** **Names and sizes of beta- and gamma-herpesvirus LTFs**. The name and size of each LTF from each of the six viruses are listed and correlated with a generalized nomenclature for LTFs.

	HCMV (β)		MCMV (β)		EBV (γ)		HHV6 (β)		HHV7 (β)		KSHV (γ)	
**LTFa**	UL49	570	M49	536	BRFR2	591	U33	470	U33	477	ORF66	429
**LTFb**	UL79	295	M79	258	BVLF1	272	U52	258	U52	254	ORF18	257
**LTFc**	UL87	941	M87	926	BcRF1	750	U58	772	U58	775	ORF24	752
**LTFd**	UL91	111	M91	134	BDLF3.5	77	U62	87	U62	75	ORF30	77
**LTFe**	UL92	201	M92	230	BDLF4	225	U63	218	U63	211	ORF31	194
**LTFf**	UL95	533	M95	417	BGLF3	332	U67	343	U67	346	ORF34	327
**Total**		2651		2501		2247		2148		2138		2036

## Data Availability

Data is contained within the article or Supplementary Material.

## Supplementary Materials

The following supporting information can be downloaded at: https://www.mdpi.com/article/10.3390/v17060779/s1, Supplementary Figures.pdf: Figure S1. Effect of LTFf phosphorylation on the EBV LTF complex, Figure S2. Buried surface areas of LTF complexes, Figure S3. Structural predictions of KSHV LTFa and LTFe with and without Zn and promoter DNA, Figure S4. Structural predictions of HHV7 LTFa and LTFe with and without Zn and promoter DNA. Data File S1.pdf: Sequence comparisons for the LTFs from HCMV, MCMV, EBV, KSHV, HHV6, and HHV7. Data File S2.pse: PyMOL session showing predicted structures of LTF complexed from HCMV, MCMV, HHV6, HHV7, EBV, and KSHV. Data File S3.pse: PyMOL session showing HHV7 LTF complex with extracted subunits. AF3color.py: PyMol script to color structures with AlphaFold 3 confidence colors.
