# Supplementary material for: Structure Prediction of Complexes Controlling Beta- and Gamma-Herpesvirus Late Transcription Using AlphaFold 3
Source: Viruses. 2025 May 29;17(6):779. doi: 10.3390/v17060779 (PMC12197705; doi:10.3390/v17060779)
Supplement: Supplementary file 1 [file viruses-17-00779-s001.zip › Supplemental Data File S1.pdf]

Sequence comparisons for the LTFs from HCMV, MCMV, EBV, KSHV, HHV6, and HHV7

For each viral LTF, Identical Protein Groups in the NCBI webserver was queried to obtain the sequence of the factor most highly represented in each virus type. The constraint-based multiple alignment tool (COBALT) (https://www.ncbi.nlm.nih.gov/tools/cobalt/cobalt.cgi?CMD=Web) was used to compare homologous LTFs across the six virus types. Because the nomenclature is different for each type of virus the LTFs were named LTFa through LTFf as shown in the table below:

LTF	HCMV	MCMV	EBV	KSHV	HHV6/HHV7
**LTFa**	UL49	M49	BRFR2	ORF66	U33
**LTFb**	UL79	M79	BVLF1	ORF18	U52
**LTFc**	UL87	M87	BcRF1	ORF24	U58
**LTFd**	UL91	M91	BDLF3.5	ORF30	U62
**LTFe**	UL92	M92	BDLF4	ORF31	U63
**LTFf**	UL95	M95	BGLF3	ORF34	U67

The representative sequences for each LTF are provided below followed by their COBALT alignment.

LTFa

>HCMV
MANRRLRHAPHTATDEFHQALCRLFAPLCVHEDHFHVQLVIGRGALQPEEAAVETSQPPAQFAAQTSAVLQQQLVHHVPRSCVLHLFVT
DKRFLNRELGDRLYQRFLREWLVCRQAEREAVTALFQRMVMTKPYFVFLAYVYSMDCLHTVAVRTMAFLRFERYNTDYLLRRLRLYPPE
RLHALLDGVTASLLGDLHRFLFGVDLRLPVLHPTSSPCLALLRAKRFDARADLAVYHRNQWCHQRQPRSPQLRGLIAALRRHAGKVPCG
NPLYVLARQAVQTFCDTCPRYLVPLRALGLHDETRGGGSTAAAAAVGHAGAGQQARHVEPTKIVLFALSAALRGGLIGSVIDLPLWCLC
RLKCERHLDARSLVAVVCRQCGHCLNLGKEKLHCQQNFPLNSMFYYRDRQEKSVIFNTHAELVHCSLCGSQRVVRQRVYELVSETLFGQ
RCVRVGWKAVLGLNAACAVYDHRLAFDVILPCAARTCDSTVVVRGVTVPRLLRLTSHGHGLLCARCQTGEYRDSCLESEDGAPLCRGCA
LVKQTACHVGGHIVQQARGGLAAISSSSSPHGLPHV

>MCMV
MRPVRRVIRELFGGVCQHRDKYHLQLLLSGTVEDERDGRRVYCVLNMFMNGKRFLCKDVVDELYCRFLERWSSCSALVKSIVREISQKE
VMTRHVFVLLAYFYAVRCMERVSRNVSCLYLRSGMSSRLEHVFKRYSRSKVDALVDNASYQGLTDLHQFVFHFPFGIPIPNQASSPCVA
FLRAREYEVGCDLPVYHRRLNLRRLGPDPGVSTLVSILRERCRETPCGNPFYVMAKVFVERYCRRRPRFLIPIGNRTLRYGGRAAVRTS
DPGGGGGGSGNSAATATDGTNCPWPLSKLSTFATSVVLRNGLISSLIDLPVWCFCKTKCQRYAEDGVLEAILCDNCGHCLNMGKDKLEG
NHTFALNCIFYYRDRQEKSVIYSTHNDTAHCSLCGNQYLSRERIYEVTRADYHHGLRFATVRWRAVIGSNAACGVLGPGTRLDVLVPCS
SRTCFGTVVLRESSVEKLLRLVSHSGEFFCQSCQNVYRETCLDRDDLDLADACLGCQIHSRCSCLEIRRRNYNNNNTGAAGPINTIITE
LN

>EBV
MALFLARHTLSGTGAGGHGRGPAPDVSEVDLTLPALGEREFSRLLDLGLACLDLSYVEMREFVVWGRPPASEAAVASTPGSLFRSHSSA
YWLSEVERPGGLVRWARSQTSPSSLTLAPHLGPSLLSLSAFTGGGCGAVAFCNAFFLAYFLVVRSVFPAFSDRIAAWICARSPFCENTR
AVARGYRGLVKRFLAFVFERSSYDPPLLRQNSRPVERCFAIKNYVPGLDSQSCVTVPSFSRWAQSHASELDPREIRDRVTPATAPSFVA
DHASALLASLQKKASDTPCGNPIQWMWYRLLVNSCLRSAHCLLPIPAVSEGGRKTGGGVGEELVGAGGPCLSRDVFVAIVSRNVLSCLL
NVPAAGPWAYKCFRSLASRPVSGPDYPPLAVFCMDCGYCLNFGKQTGVGGRLNSFRPTLQFYPRDQKEKHVLTCHASGRVYCSNCGSAA
VGCQRLAEPPSARSGWQPRIRAVLPHNAAYELDRGSRLLDAIIPCLGPDRTCMRPVVLRGVTVRQLLYLTLRTEARAVCSICQQRQAPE
DARDEPHLFSSCLEVELPPGERCAGCRLYQTRYGTPAAQAHPPGEAGGGFSRQSPAS

>KSHV
MALDQRWDRFLVSWFGLDEAQLTAHRVFEGENGVPVEEYVAFVIFGERGFQGNMPSWARHLLDRPSLAQAIAVLRAGSDTVAKQAQICA
AQQLLGAHVWVVVTLSRAQAADHARAIPRHVWAKYLSLPFSKACAQLCKLLALCSRFPLVTCCSKPPPSLPWLRKKWHGPLPRRPLLEV
PSPTRRGVAATEDGNGLGIGAADTGLREALERVAPTVPCGNPFDAMLGSLCFLSLIKSRHVVLPACEQEGPGLVRNLGRRLLAYNVLSP
CVSIPVICSRVARAALAKRARCARAVVCMECGHCLNFGRGKFHTVNFPPTNVFFSRDRKEKQFTICATTGRIYCSYCGSEHMRVYPLCD
ITGRGTLARVVIRAVLANNAALAIRDLDQTVSFVVPCLGTPDCEAALLKHRDVRGLLQLTSQLLEFCCGKCSS

>HHV6
MFYLKKLLRQLLAPLCKHGPYTHLQLFVMGDACVPGKCVLTMFLTNKKFLNKEVTEKFYNEFFAIWLRCRPETRFITKRLFNKMVMTKG
LFVLLAYLYFVYRQCKVLELLSLYKLKTIKWMDVETRFRVYPSYKLNKLLEMPSFSEINELHMFLFEQQLLLPIPTHVNLPCMRLFCLR
DYEQTETVMLRYRQREHVLSFPSMLQKYALKSPAGNFMFTMAKALVENFCFSADRYLIPVEHNNLVPMVPSKPERGDFPKILTFALATS
LKDGLATSVISLPVMCYCKTKCSRFILEESYICVICAKCGHCLNSGKEKLCSPQGFSLSSMFYFRDKQEKNLIYSMHTDVMYCSLCGSQ
QLVFERIYEMSEHCVLGMKVKTVSWKAVIGTNSACTILNDNVKFDVIVPCSCRSCYSTVHLYNVTVKKLLRLVSHGSDFQCQHCQHSFR
ETCLDLEDCVNICQGCQISQNVRCI

>HHV7
MSSLRNLLCRLISPLCKHGSYSHLQLFLIGDVSKSENCVLSMFVTNKKFLSKDLTDNFYRKFLKVWLKCDLDSRNQIKAIFNKMMMTKN
FFLLLAYLYFLYRQCKVLKILALYKIQRLTWKQIVERFKQYPIHKLNQLLEIPSFVSFNDLYYYLFQQQLMLPISTHCNIPCMKLFCLR
NFEQCNDITLRYAHRASNLTYQDIFHGCSLTVPCGNFIFAMAMAMIENYCYSFDRFLIPLENNNLVPIVLNKQEKHQLPKILTFALTTV
LKRSLTSSIISLPVLCFCKTKCLRYAKIDSYLTVICSKCGHCLNSGKERLKGKQTFSLSSMFYYRDRQEKNIIYSMHTDLLYCSLCGGQ
RLTLEKVYELYEYSVSGIQVKSVSWKAIIGTNSACTILNDSVKFDAIVACSCRTCYSMIHLQNLTINKLLKLISHSTEFQCQDCQNIYR
ETCLDLEDCGEICTGCKISQLAKCTQHGCDTW

COBALT analysis of LTFa

Bold residues below are involved in Zn binding.

```
HCMV  1    MANRR[13]QA   LCRLFAPLCVHEDHFHVQLVIGRGALQPEEAAVET[6]FAAQTSAVLQQQLVHHVPRsCVLHLFVT  89
MCMV  1    MRPVR    RV   IRELFGGVCQHRDKYHLQLLLSGTVEDERDG----   -----------RRVY-----CVLNMFMN  50
EBV   1    MALFL[13]GR[5]VSEVDLTLPALGEREFSRLLDLGLACLDLSYVEMR[1]FVVWGRPPASEAAVASTPG----SLF--  83
KSHV  1    -----    --   --------MALDQRWD-RFLVSWFGLDEAQLTAHR[1]FEGENGVPVEEYVAFVIFG---------  46
HHV6  1    MFYLK    KL   LRQLLAPLCKHGPYTHLQLFVMGDACVPGK-----   --------------------CVLTMFLT  45
HHV7  1    MSSLR    NL   LCRLISPLCKHGSYSHLQLFLIGDVSKSEN-----   --------------------CVLSMFVT  45

HCMV  90   DKRFLNRELGDRLYQRFLREWLVCRQAEREAVTA   LFQRMVMTKP   YFVFLAYVYSMDCLHTVAVRTMAFLRFERY  163
MCMV  51   GKRFLCKDVVDELYCRFLERWSSCSALVKSIVRE   ISQKEVMTRH   VFVLLAYFYAVRCME-RVSRNVSCLYLRSG  123
EBV   84   RSHSSAYWLSEVERPGGLVRWARSQTSPSSLTLA[6]LLSLSAFTGG[8]NAFFLAYFLVVRSVFPAFSDRIAAWICARS  171
KSHV  47   ----------ERGFQGNMPSWA------RHLLDR[2]LAQAIAVLRA[8]AQICAAQQLLGAHVWVVVT-------LSRA  107
HHV6  46   NKKFLNKEVTEKFYNEFFAIWLRCRPETRFITKR   LFNKMVMTKG   LFVLLAYLYFV-YRQCKVLELLSLYKLKTI  118
HHV7  46   NKKFLSKDLTDNFYRKFLKVWLKCDLDSRNQIKA   IFNKMMMTKN   FFLLLAYLYFL-YRQCKVLKILALYKIQRL  118

HCMV  164  NTDYLLRRLRLYPPERLHALLDGVTASLLGDLHRFLFGVDLRLPVLHPT    SSPCLALLRAKRFDARADLAVYHRNQW  239
MCMV  124  MSSRLEHVFKRYSRSKVDALVDNASYQGLTDLHQFVFHFPFGIPIPNQA    SSPCVAFLRAREYEVGCDLPVYHRRLN  199
EBV   172  PFCENTRAVARGYRGLVKRFLAFVFERSSYD--PPLLRQNSR-PVERCF[10]SQSCVTVPSFSRWAQSHASELDPREIR  254
KSHV  108  QAADHARAIPR---HVWAKYLSLPFSKACAQLCK-LLALCSRFPLVTCC[ 1]-----KPPPSLPWLRKKWHGPLPRRPL  175
HHV6  119  KWMDVETRFRVYPSYKLNKLLEMPSFSEINELHMFLFEQQLLLPIPTHV    NLPCMRLFCLRDYEQTETVMLRYRQR-  193
HHV7  119  TWKQIVERFKQYPIHKLNQLLEIPSFVSFNDLYYYLFQQQLMLPISTHC    NIPCMKLFCLRNFEQCNDITLRYAHR-  193

HCMV  240  CHQRQPRSP    QLRGLIAALRRHAGKVPCGNPLYVLARQAVQTFCDTCPRYLVPLRALGLHDETRGGGSTA[6]HAGA  318
MCMV  200  LRRLGP-DP    GVSTLVSILRERCRETPCGNPFYVMAKVFVERYCRRRPRFLIPIGNRTLRYGGRAAVRTS[6]GGSG  277
EBV   255  DRVTPATAP[ 5]HASALLASLQKKASDTPCGNPIQWMWYRLLVNSCLRSAHCLLPI------PAVSEGGRKT   GGGV  326
KSHV  176  LEVPSPTRR[15]ADTGLREALERVAPTVPCGNPFDAMLGSLCFLSLIKSRHVVLPA----------------   ----  243
HHV6  194  --------E    HVLSFPSMLQKYALKSPAGNFMFTMAKALVENFCFSADRYLIPVEHNNLVPMVPS-----   ----  249
HHV7  194  --------A    SNLTYQDIFHGCSLTVPCGNFIFAMAMAMIENYCYSFDRFLIPLENNNLVPIVLN-----   ----  249

HCMV  319  GQQARHVEP   TKIVLFALSAALRGGLIGSVIDLPLWCLCRLKCERHLDARSL   VAVVCRQCGHCLNLGK   EKLH  389
MCMV  278  NSAATATDG[7]SKLSTFATSVVLRNGLISSLIDLPVWCFCKTKCQRYAEDGVL   EAILCDNCGHCLNMGK   DKLE  355
EBV   327  GEELVGAGG   PCLSRDVFVAIVSRNVLSCLLNVPAAGPWAYKCFRSLASRPV[7]LAVFCMDCGYCLNFGK[5]GRLN  409
KSHV  244  ----CEQEG   PGLVRNLGRRLLAYNVLSPCVSIPVICSRVARAALAKRARCA   RAVVCMECGHCLNFGR   GKFH  310
HHV6  250  --KPERGDF   PKILTFALATSLKDGLATSVISLPVMCYCKTKCSRFILEESY   ICVICAKCGHCLNSGK   EKLC  318
HHV7  250  --KQEKHQL   PKILTFALTTVLKRSLTSSIISLPVLCFCKTKCLRYAKIDSY   LTVICSKCGHCLNSGK   ERLK  318

HCMV  390  CQQNFPLNSMFYYRDRQEKSVIFNTHAELVHCSLCGSQRVVRQRVYELVSETLF-GQRCVRVGWKAVLGLNAACAVYDHR  468
MCMV  356  GNHTFALNCIFYYRDRQEKSVIYSTHNDTAHCSLCGNQYLSRERIYEVTRADYHhGLRFATVRWRAVIGSNAACGVLGPG  435
EBV   410  ---SFRPTLQFYPRDQKEKHVLTCHASGRVYCSNCGSAAVGCQRLAEPPSARSG-----WQPRIRAVLPHNAAYELDRGS  481
KSHV  311  -TVNFPPTNVFFSRDRKEKQFTICATTGRIYCSYCGSEHMRVYPLCDITGRGTL-----ARVVIRAVLANNAALAIRDLD  384
HHV6  319  SPQGFSLSSMFYFRDKQEKNLIYSMHTDVMYCSLCGSQQLVFERIYEMSEHCVL-GMKVKTVSWKAVIGTNSACTILNDN  397
HHV7  319  GKQTFSLSSMFYYRDRQEKNIIYSMHTDLLYCSLCGGQRLTLEKVYELYEYSVS-GIQVKSVSWKAIIGTNSACTILNDS  397

HCMV  469  LAFDVILPCAAR--TCDSTVVVRGVTVPRLLRLTSHG-HGLLCARCQTgEYRD    SCLESEDG--APLCRGCALVKQT  539
MCMV  436  TRLDVLVPCSSR--TCFGTVVLRESSVEKLLRLVSHS-GEFFCQSCQN-VYRE    TCLDRDDLdlADACLGCQIHSRC  507
EBV   482  RLLDAIIPCLGPdrTCMRPVVLRGVTVRQLLYLTLRTeARAVCSICQQrQAPE[10]SCLEVELPp-GERCAGCRLYQTR  566
KSHV  385  QTVSFVVPCLGTp-DC-EAALLKHRDVRGLLQLTSQL-LEFCCGKCSS-----    -----------------------  429
HHV6  398  VKFDVIVPCSCR--SCYSTVHLYNVTVKKLLRLVSHG-SDFQCQHCQH-SFRE    TCLDLEDC--VNICQGCQISQNV  467
HHV7  398  VKFDAIVACSCR--TCYSMIHLQNLTINKLLKLISHS-TEFQCQDCQN-IYRE    TCLDLEDC--GEICTGCKISQLA  467

HCMV  540  ACHVGGHIVQQARGGLAAISSSSSPHGLPhv  570
MCMV  508  SCLEIRRRNYNNNNTGAAGPINTIIITELN--  536
EBV   567  YGTPAAQAHPPGEAGGGFSRQSPAS------  591
KSHV       -------------------------------
HHV6  468  RC---------------------I-------  470
HHV7  468  KC---------------------TQHGCDtw  477
```

6% identity overall

LTFb

```
>HCMV
MMARDEENPAVPRVRTGKFSFTCANHLILQISEKMSRGQPLSSLRLEELKIVRLICVLLFHRGLETLLLRETMNNLGVSDHAVLSRKTP
QPYWPHLYRELRQAFPGLDFEAAVFDETRAARLSQRLCHPRLSGGLLTRFVQRHTGLPVVFPEDLARNGNILFSLGTLYGHRLFRLAAF
FTRHWGAEAYEPLIRIICQKMWYFYLIGTGKMRITPDAFEIQRSRHETGIFTFIMEDYRTFAGTLSRHPHRPHPQQQQHHHPGPPHPPL
SHPASSCLSPEAVLAARALHMPTLANDV

>MCMV
MVKTIRVGRFLHLSDDNHLILHITTKLLSGQPLSSMRLEELKIIRLACLLTLGRGIELLILRETVANNGVSDNTILNRKISPEFWRKMY
EAMRAHVPTETLHRAFSERSAAALSVEMTGSRACRALVSHLIRTETGLALSLPDELLSDGNIFFSLGTVYGHRLFRLLRFFNRHWGKEA
HEPAIRTICQKVWFFYLIAWKKLTVSPEAFSVQRSDHELGIFSFLIQDYLTFTGTLRRSTPPMDKKEEGVIADLLSGALE

>EBV
MLMGLGVRGDDARMTPGIHGLLLKMLKNWPLCALREDELRFLHLSLTKLLTLSVNFYLWREAVINTGNRTNRVLARKVPDEYWYLLYRA
LARVGFPAEALRPGNRSRSLCLFLHDRPDVTGAVCACVWAQLGLRRAPDLSQVTLADGNLLFNLGSVLPNRLVVGVLYCLVHWGADEHE
TRVRARLRPLFVAFLCLAGYLLLDRAILSDAHDYEGLWHAVALSMAAWHGLTPLPETRDEKEAKPPCDDFIYLFANDPLQCEELAQLGA
TGEAR

>KSHV
MLGKYVCETEPLSPGLRRLMWRFLQNKNLNTFHAQELRFIHLVLCKMYNFGLNVYLLREATANAGTYDEVVLGRKVPAEVWKLVYDGLE
EMGVSSEMLLCEAYRDSLWMHLNDKVGLLRGLANYLFHRLGVTHDVRIAPENLVDGNFLFNLGSVLPCRLLLAAGYCLAFWGSDEHERW
VRFFAQKLFICYLIVSGRLMPQRSLLVWASETGYPGPVEAVCRDIRSMYGIRTYAVSGYLPAPSEAQLAYLGAFNNNAV

>HHV6
MTQIGQYIKLNDAVPNLILHITTKLLRNENLTSFKQEELLLIQHVCTSMLSHGIKILLLRESLYNSGIGDIVILNRKISNNYWFRLFSI
LKQHSDAELLRHMFNESHSAYISKKLHYSGNVSHIINFLFMDEFGLSLKIPEEIICEGNIVFSVGAIYNHRLLKICRFFNRFWGDQERE
PAVRLICKHLWFAYLIMFGKFEISTLAYNQQRAEHKAGLFSFLQNDFKVFCGMSENPQFLDSSAIFDLTGISAEDLFSYE

>HHV7
MTQVGQYIISNNSTSNLILHITKKLVSGESLFNLKQEEILIIQNVCTLMFSHGIQILLLRETLHNIGVSDLVVLNRKVPDEFWFKIFCM
IKQRSGSEILKHIFSEENAAQLSKKLHHTGIVKQIIESFFLDEFGLSITIPAEIIHDGNIMFSIGAIYNHRLLKLCRYFNKFWGQEVYE
PFIRKICKHLWFGYLIFFDKIKISHGAFSQQKPEHRNGLFTFIQNDFKVFCGIVEKEAKVTECNLSDLFSIGPLQF
```

COBALT analysis of LTFb

```
HCMV   1     [12]RVRTGKFSFTCAN---HLILQISEKMSRGQPLSSLRLEELKIVRLICVLLFHRGLETLLLRETMNNLGVSDHAVLS  85
MCMV   1     [ 3]TIRVGRFLHLSDD--NHLILHITTKLLSGQPLSSMRLEELKIIRLACLLTLGRGIELLILRETVANNGVSDNTILN  77
EBV    1         -MLMGLGVRGDDArMTPGIHGLLLKMLKNWPLCALREDELRFLHLSLTKLLTLSVNFYLWREAVINTGNRTNRVLA  75
KSHV   1         --MLGKYVCETEP-LSPGLRRLMWRFLQNKNLNTFHAQELRFIHLVLCKMYNFGLNVYLLREATANAGTYDEVVLG  73
HHV6   1         MTQIGQYIKLNDA-VPNLILHITTKLLRNENLTSFKQEELLLIQHVCTSMLSHGIKILLLRESLYNSGIGDIVILN  75
HHV7   1         MTQVGQYIISNNS-TSNLILHITKKLVSGESLFNLKQEEILIIQNVCTLMFSHGIQILLLRETLHNIGVSDLVVLN  75

HCMV   86    RKTPQPYWPHLYRELRQAfpGLDFEAAV--FDETRAARLSQRLCHPRLSGGLLTRFVQRHTGLP---VVFPEDLArNGNI  160
MCMV   78    RKISPEFWRKMYEAMRAH---VPTETLHRAFSERSAAALSVEMTGSRACRALVSHLIRTETGLA--LSLPDELLS-DGNI  151
EBV    76    RKVPDEYWYLLYRALARV--GFPAEALR---PGNRSRSLCLFLHDRPDVTGAVCACVWAQLGLRraPDLSQVTLA-DGNL  149
KSHV   74    RKVPAEVWKLVYDGLEEM--GVSSEMLL---CEAYRDSLWMHLNDKVGLLRGLANYLFHRLGVThdVRIAPENLV-DGNF  147
HHV6   76    RKISNNYWFRLFSILKQH--S-DAELLRHMFNESHSAYISKKLHYSGNVSHIINFLFMDEFGLS--LKIPEEIIC-EGNI  149
HHV7   76    RKVPDEFWFKIFCMIKQR--S-GSEILKHIFSEENAAQLSKKLHHTGIVKQIIESFFLDEFGLS--ITIPAEIIH-DGNI  149

HCMV   161   LFSLGTLYGHRLFRLAAFFTRHWGAEAYEPLIRIICQKMWYFYLIGTGKMRITPDAFEIQRSRHETGIFTFIMEDYRTFA  240
MCMV   152   FFSLGTVYGHRLFRLLRFFNRHWGKEAHEPAIRTICQKVWFFYLIAWKKLTVSPEAFSVQRSDHELGIFSFLIQDYLTFT  231
EBV    150   LFNLGSVLPNRLVVGVLYCLVHWGADEHETRVRARLRPLFVAFLCLAGYLLLDRAIL--SDAHDYEGLWHAVALSMAAWH  227
KSHV   148   LFNLGSVLPCRLLLAAGYCLAFWGSDEHERWVRFFAQKLFICYLIVSGRLMPQRSLLVWASETGYPGPVEAVCRDIRSMY  227
HHV6   150   VFSVGAIYNHRLLKICRFFNRFWGDQEREPAVRLICKHLWFAYLIMFGKFEISTLAYNQQRAEHKAGLFSFLQNDFKVFC  229
HHV7   150   MFSIGAIYNHRLLKLCRYFNKFWGQEVYEPFIRKICKHLWFGYLIFFDKIKISHGAFSQQKPEHRNGLFTFIQNDFKVFC  229

HCMV   241   GT[6]RPHPQQQQHHHPGPPHPPLSHPASSCLSPEA[16]   295
MCMV   232   GT[1]RRSTPPMDKKEEG-VIADLLSGALE------       258
EBV    228   GL   TPLPETRDEKEAKPPCDDFIYLFANDPLQCE[12]   272
KSHV   228   GI   RTYAVSGYLPAPSEAQLAYLGAFNNNAV---       257
HHV6   230   GM   ----SENPQFLDSSAIFDLTGISAEDLFSYE       258
HHV7   230   GI   ----VEKEAKVTECNLSDLFSIGPLQF----       254
```

9% identity overall

LTFc

>HCMV
MAGAAPRRLGCDALIVVGGSAMPRRVLHVPVHVRACNLTQELSTGEDARFCRPRPVNVERVRAVFAALYRACPIHVRTEPERVKLVLGR
LLLGPVAVPCFCDGEVEGHGEHLVPTTQFCRGPLLYVHRRCCCGSVTAGRALSYHVLENHVATHVLRGLLSLTEWNRELPSLFCDCPGG
GGASGTEERYAMACLPRDLSLHLDDYPYLMVEIGRVLSVSEVDDYVTAVSGYLGEAAAPRIQVHYKLLFGLNVRPQAPCALDATRDFFL
LELQKLWLGVEYHHEVTSEFFGRVLAQLHRDRARVMMALRLPEQTVCHLSTFVLSRFKRQVLYFKLQVSYGKCRTGHADRSGGGGNGGN
QGHHNLLCYRRLSVTFADTDTVWRNLFYVYYELARDLGSHGTENRPVNRGYGVSCAPRTSRLSPSESTVVSANGHALSSTALPTTSAGH
KLSLPRDPAADRVRRYVCIISRLMYARYGERWRKHRQRRSETGEEEEEETLESGETDATPPFDFTGQQLRRAYQEHRRRKHLAVQRYAP
CRRKLIGGMEFAEVTGVSLDRIAVNAFNTNRVINMKAALSSIAASGLGVRAPRLPKNMTHSFVMYKHTFKEPACTVSTFVSNDAVYINS
LNVNIRGSYPEFLYSLGVYRLHVNIDHFFLPAVVCNSNSSLDVHGLEDQAVIRSERSKVYWTTNFPCMISHTNNVNVGWFKAATAIVPR
VSGADLEAILLKELSCIKNMRDVCIDYGLHRVFTQLELRNSYQIPFLAKQLVLFLRACLLKLHGREKRLQLDRLVFEAAQRGLFDYSKN
LTAHTKIKHTCALIGSRLANNVPKILARNKKVKLDHLGRNANVLTVCRHVEAHKIPRTRLKVLVEVLGVLQSISGTPHTREVIHQTLFR
LCSAAAATSGLCSSPPPLCVSSSSSVPSVPTSVSVDGSSEPTSPRARFASR

>MCMV
MMSASDGSPASLSCLDPALIVKSPTARVKSAPVCVNSYNLTREISPFEDSRLSQAVTVDEEHISSIFRTLMAAGPDPGATDEDKARVVL
CRLLMGPVAVPCYCDEWDVDDYLAKCAYRCSGPALYVHRSRCRCGAEGGGTMFTLLHDHYTTHVFRGLLSLSEWNVRLTDVFCACNAFR
SDRYVMAVLPKHQSVFIEYYPYFLVCLARYLTVPEIDDCANSMTAHLGPAIAARVGVHYKMLFGANARPPRVTEVARRANYDLFLLELQ
KLWLNVSYRNAVTRDFFETVFSAFHHETGKVMLALRSPGRQPLFPRWISMSRFKKQVLYFELEVRCTKSRKDELKNALIFRKTSVLFAD
HDVIWRNLFYTYYAWCAHRGFGGESRLWGPSGSGGGAATESERGGGVRGRDQATTASAAAAAASTDDDASAASTESGVAVSAPAAAATS
AAKAHPHAPAAPTVVGGGQGTAVSSTATQKYVKIVDRLALVRLRFERERRLAAEAAVGSGSGSVEGAVAGGTSGVQTDVKADEPVAVPSF
DFDPYRCLVRHRAAEGVRREGEGSVYGARKIIGGREFSEMTAVSLNRVAVNAFNTNRVINLKATIVQTPRLSAFYVPRNMTHSFVMYKH
TFKEPPYTVSTFVSNDAAHTNSLNVNIRGSYQEFLYALSVYKLYVNIENFFLPASVCNSNSSLDVHGIEDQGVIRSERDKVYWTTNFPC
MISNTDNINVGWFKAATAIIPKVSGVALENVLLKELAYVTSIDQLCVDYTLHRVFTVLETRNCYQIPFLSKQFILFVRIMMLRICGLEH
RLAVDRLIFRAIRQGVFDYHKNTVAHTKIKHTCALVGTRLANNVPKVLVKNKKIKLDYLGRNANLLTLCRHVDHACVDAHRLEALIGVL
DCLEKLTSIDRTKEALTRARVRLCGGYRPEAATSRR

>EBV
MTQGKREMGGGLEGFSSQLGLFYALACNRSPPALPEDATLLIKWLDTALGREATFYACRAMRRLLLGVIRINDCQELPPGLIILSPGTV
PGPLGVQSLEHTDCEIWSSAHPDHAAHLPVPRVITYTDCPGSISTSSMFRLIIRYLSHHQFERCFEQFCRVVPRRFLGTCKQNSAKMLA
HLKQVTRIPPCPPFSGREARLKFHFFSWSTFMLSWPNNATLREIRTRAATNLTHHPHLVDTLYHASPQTPFLTRSGALYRFVTCCNCTL
PNISIQQCKAGDRPGDLEIILQSNGGGRPASFQFPSSPTGALLRCIVAAALLPEVSVGHQELSPLMSRSHGGQTDVRSGPDPARRLVAL
LRREDGAPKDPPLGPFGHPRGPGPAKSEDEESERRDAPPPPLDFSFQASRLVPVGPGFRLLVFNTNRVINTKLVCSEPLVKMRVCNVPR
LINNFVARKYVVKETAFTVSLFFTDGVGANLAINVNISGTYLSFLLAMTSLRCFLPVEAIYPAAVSNWNSTLDLHGLENQSLVRENRSG
VFWTTNFPSVVSCQDGLNVSWFKAATATISRVHGRTLEQHLIREITPIVTHREAKISRIKNRLFTLLELRNRSQIQVLHKRFLEGLLDC
ASLLRLDPSCINRIASEGLFDFSKRSIAHSKNRHECALLGHRHSANVTKLVVNERKTRLDILGRNANFLTRCKHQVNLRQSPIFLTLLR
HIRRRLGLGRASVKREITLLLAHLRKKTAPIHCRDAQV

>KSHV
MAALEGPLLLPPSASLTTSPQTTCDQATWESQLEIFCCLATNSHLQAELTLEGLDKMMQPEPTFFACRAIRRLLLGERLHPFIHQEGTL
LGKVGRRYSGEGLIIDGGGVFTRGQIDTDNYLPAVGSWELTDDYDKPCEFRELRSLYLPALLTCTICYKAMFRIVCRYLEFWEFEQCFH
AFLAVLPHSLQPTIYQNYFALLESLKHLSFSIMPPASPDAQLHFLKFNISSFMATWGWHGELVSLRRAIAHNVERLPTVLKNLSKQSKH
QDVKVNGRDLVGFQLALNQLVSRLHVKIQRKDPGPKPYRVVVSTPDCTYYLVYPGTPAIYRLVMCMAVADCIGHSCSGLHPCANFLGTH
ETPRLLAATLSRIRYAPKDRRAAMKGNLQACFQRYAATDARTLGSSTVSDMLEPTKHVSLENFKITIFNTNMVINTKISCHVPNTLQKT
ILNIPRLTNNFVIRKYSVKEPSFTISVFFSDNMCQGTAININISGDMLHFLFAMGTLKCFLPIRHIFPVSIANWNSTLDLHGLENQYMV
RMGRKNVFWTTNFPSVVSSKDGLNVSWFKAATATISKVYGQPLVEQIRHELAPILTDQHARIDGNKNRIFSLLEHRNRSQIQTLHKRFL
ECLVECCSFLRLDVACIRRAAARGLFDFSKKIISHTKSKHECAVLGYKKCNLIPKIYARNKKTRLDELGRNANFISFVATTGHRFAALK
PQIVRHAIRKLGLHWRHRTAASNEQTPPADPRVRCVRPLV

>HHV6
MQHTGNCETLIVNSCFGSTCARSIPVFIDSCDLTAEVSRDEETRLARSVPVVLEKIESIIEKIFQTSGPNIVHDKDRAKIALCRLLLGP
VAVPCFCEEWDTNDYLSKSGCKCLGPILYIHTSRCRCSDIPVFKFSIMKDYYASHVFRGLLSLKEWNTHLPNVLCTCELSMSDRYVATV
FPKQNSIYLEYYPYFLCYLCRYLTVIEIEQCTNDLISLLGPKVAQRVIIHFKLLFGFRHKPHMGTVDSWFWENFFMLELHKLWLTVVKH
NRVTTDFFNVVYEKIQNYKQYAIKTLRMSSKAVPAIQRFCLAKFKQQLLYLNIKVTVKKNKRELCLNGFVYGKTLYVVESSQLIFRNLL
LLYYDYSLPGECKTNEENVLTTHYIRVISRLSFKQSRSAVPPGVRPDFTFVAQLPKRKELPNVPGGIDFAEITSVRHGAVILNAFNTNK
VMNLKATISKRANFVYHRIPKTMTHSFVMYKHTFKEPAFTVSTFVSNDDLDMSSLNINIRGPYCDFLYALGVYKMHVSIQDLFLPAFVC
NSNNSVDLQGLEDQDVVRNRKKKVYWITNFPCMISNANKVNVGWFKAGTGIIPRVSGKDLQNVLLQELNNVREIPGLVFDMDLHQLLVL
LEQRNLHQIPFLVKQFLIFLRLGLLMGYGHSRRNKVHDIMLHLISNGLFDFNKNSVANTKIKHGCALVGTRLANNVPKIIARQKKMKLD
HMGRNANSLAVLRFIVKSGEHKNKTVFIKLLEYLAETSTAINTRNEVARLLQTLTTNMKT

```
>HHV7
MIDSISEETLIVKSYTVNHCAKNVPVFINSYDLTAEVAKNEDVRLARQVQISLEKIDEVIESIFSASGPSVENVKDQAKFALCRLLLGP
VSIPCYCEEWDVNFYLTKCSYNCEGPVLYIYKNASQCCESTYRFSIMTNYHSTHIFRGLLSLQEWNSHLSNILCTCSNVTGDKYTATIF
PNNASIYLEYYPYFLCYLCKHLSIIDIEQCTNELIAFLGPKTSQRIIIHYKLLFGFRSKPMNFTVSLLEQVFTLEIQKLYYSVSKHNST
TADFFNVITAKFAEDKYFVLRTFKLSAQITPGIQSFCSLKFKLQTLYLNLKIMKNTKLSISNSFYHGKTLYTLDEKQLVWRNLLLIYYG
YNLKDNVKQTQEESLLSMHYIRILERLSLKSFREINQQFSFEIPSYQEKTLQFIPGGNDFAEITSVTHGETTVNAFNTNRVMNVKAALS
GEIHCVLHRIPKSMTHSFVMYKRTFKEPSLTVSTFISNDDFTTSSLNINIRGPYCDFLYALGVYRLHVNIQDFFLPAFVCNSNNSMDLH
GLENQGIVRKRKKKVYWITNFPCMISNSEKVNVGWFKAGTGIIPKVSGTDLKNVLLKELISIGEIPNITFDMDLHALLTLLEKRNMHQV
PFLIKQFFMFLRLGLLVGYGRKQERKVHHIMLFLIQKGFFDFSKNSVANSKIKHACALVGSRLANNVPKILSKQKKMKLDHLGRNANAL
TVLRFIVENGYYKRKTIFRKLLKYLATTSFNAHVQTESNRLLNLMHNDSKTNFSSLERLYTLR
```

COBALT analysis of LTFc

```
HCMV   1     MAGA---APRRLGC--DALIVVG--GSAMPrrvLHVPVHVRACNLTQELSTGEDARFCRPRPVNVERVRAVFAALYRACP  73
MCMV   1     MMSASdgSPASLSCldPALIVKS--PTA---rvKSAPVCVNSYNLTREISPFEDSRLSQAVTVDEEHISSIFRTLMAAGP  75
EBV    1     MTQGKreMGGGLEG------------------------------FSSQLGLFYALACNRSPPALPEDATLLIKWLDTALG  50
KSHV   1     MA--------ALEG--PLLLPPSASLTTSP------QTTCDQATWESQLEIFCCLATNSHL-----QAELTLEGLDKMMQ  59
HHV6   1     MQHTG-------NC--ETLIVNSCFGSTCA---RSIPVFIDSCDLTAEVSRDEETRLARSVPVVLEKIESIIEKIFQTSG  68
HHV7   1     MIDSI-------SE--ETLIVKSYTVNHCA---KNVPVFINSYDLTAEVAKNEDVRLARQVQISLEKIDEVIESIFSASG  68

HCMV   74    IHVRTEPERVKLVLGRLLLGPVAVPCFCDG-EVEghgEHLVPTTQFCRGPLLYVHR-RCCCGSVtAGRALSYHVLENHVA  151
MCMV   76    DPGATDEDKARVVLCRLLMGPVAVPCYCDEWDVD---DYLAKCAYRCSGPALYVHRSRCRCGA--EGGGTMFTLLHDHYT  150
EBV    51    REATFYACRA---MRRLLLGVIRIND-CQELPPG----LIILSPGTVPGPLGV----QSLEHTD-CEIWSSAHPDHAAHL  117
KSHV   60    PEPTFFACRA---IRRLLLG--------ERLHP------FIHQEGTLLGKVGRRYSGEGLIIDG-GGVFTRGQIDTDNYL  121
HHV6   69    PNIVHDKDRAKIALCRLLLGPVAVPCFCEEWDTN---DYLSKSGCKCLGPILYIHTSRCRCSDI---PVFKFSIMKDYYA  142
HHV7   69    PSVENVKDQAKFALCRLLLGPVSIPCYCEEWDVN---FYLTKCSYNCEGPVLYIYKNASQCCE----STYRFSIMTNYHS  141

HCMV   152   THVLRGLLS   LTEWNRELPSLFCDCPGGggaSGTEERYAMAC   LPRDLSLHLDDYPYLMVEIGRVLSVSEVDDYVT  225
MCMV   151   THVFRGLLS   LSEWNVRLTDVFCACN-----AFRSDRYVMAV   LPKHQSVFIEYYPYFLVCLARYLTVPEIDDCAN  219
EBV    118   P--------   --------VPRVITYTDCpgsISTSSMFRLII[7]FERCFEQFCRVVPRRFLGTCKQNSAKMLAHLKQ  182
KSHV   122   PAVGSWELT[8]FRELRSLYLPALLTCTIC-----YKAMFRIVC[7]FEQCFHAFLAVLPHSLQPTIYQNYFALLESLKH  205
HHV6   143   SHVFRGLLS   LKEWNTHLPNVLCTCE-----LSMSDRYVATV   FPKQNSIYLEYYPYFLCYLCRYLTVIEIEQCTN  211
HHV7   142   THIFRGLLS   LQEWNSHLSNILCTCS-----NVTGDKYTATI   FPNNASIYLEYYPYFLCYLCKHLSIIDIEQCTN  210

HCMV   226   AV-SGYLGEAAAPRIQVHYKLLFGLNVRPQ--APCALDATRDFFLLELQKLWLGVEYHHEVTSEFFGRVLAQLHRDRARV  302
MCMV   220   SM-TAHLGPAIAARVGVHYKMLFGANARPPRvTEVARRANYDLFLLELQKLWLNVSYRNAVTRDFFETVFSAFHHETGKV  298
EBV    183   VTrIPPCPPFSGREARLKF----------------------HFFSWSTFMLSW----PNNATLREIRTRAATNLTHHPHLV  237
KSHV   206   LS-FSIMPP-ASPDAQLHF----------------------LKFNISSFMATW----GWHGELVSLRRAIAHNVERLPTVL  258
HHV6   212   DL-ISLLGPKVAQRVIIHFKLLFGFRHKPHM-GTVDSWFWENFFMLELHKLWLTVVKHNRVTTDFFNVVYEKIQNYKQYA  289
HHV7   211   EL-IAFLGPKTSQRIIIHYKLLFGFRSKPMN-FTVS--LLEQVFTLEIQKLYYSVSKHNSTTADFFNVITAKFAEDKYFV  286

HCMV   303   MMALRLPEQTVCHLSTFVLSRFKRQV   LYFKLQVSYGKCRTGHADRSGGG[9]NLLCYRRLSVTFADTD--TVWRNLF  383
MCMV   299   MLALRSPGRQPLFPRWISMSRFKKQV   LYFELEVRCTKSRKDELK-----   NALIFRKTSVLFADHD--VIWRNLF  365
EBV    238   DTLYHASPQTPFLTRSGALYRFVTCC[6]--ISIQQCKAGDRPGDLEIILQS[4]-----RPASFQFPSSPtgALLRCIV  314
KSHV   259   KNLSKQSKHQDVKVNGRDLVGFQLAL[6]LHVKIQRKDPGPKPYRVVVSTPD   -----CTYYLVYPGTP--AIYRLVM  331
HHV6   290   IKTLRMSSKAVPAIQRFCLAKFKQQL   LYLNIKVTVKKNKRELCL-----   NGFVYGKTLYVVESSQ--LIFRNLL  356
HHV7   287   LRTFKLSAQITPGIQSFCSLKFKLQT   LYLNLKIM--KNTKLSIS-----   NSFYHGKTLYTLDEKQ--LVWRNLL  351

HCMV   384   YVYY---[18]GYGV--------[17]SANGHALSSTALPTTSAGH[11]RV   ------RRYVCIISRLMYARYG[5]  479
MCMV   366   YTYYAWC[27]GGGVRGRDQaTT[18]TESGVAVSAPAAAATSAAK[ 9]TV[8]VSSTATQKYVKIVDRLALVRL-[4]  492
EBV    315   AAALLPE    ------------    VSVGHQELSPLMSRSHGGQ    TD[7]-----ARRLVALLRREDGAPKD     366
KSHV   332   CMAVADC    ------------    IGHSCSGLHPCANFLGTHE    T-   -----PRLLAATLSRIRYAPKD     375
HHV6   357   LLYY---    DYSLPGECK-TN    -------------------    --   EENVLTTHYIRVISRLSFKQSR     393
HHV7   352   LIYY---    GYNLKDNVKqTQ    -------------------    --   EESLLSMHYIRILERLSLKSFR     389

HCMV   480   HRQRRSET   -----GEEEEEETLESGETDATPPFDFTGQQL[ 8]RRKHLAVQR[9]GGMEFAEVTGVSLD-RIAVNA  560
MCMV   493   RRLAAEAA[9]GAVAGGTSGVQTDVKADEPVAVPSFDFDPYRC[11]REGEGSVYG[5]GGREFSEMTAVSLN-RVAVNA  586
EBV    367   PPLGPFG-   HPRGPGPAKSEDEESERRDAPPPPLDFSFQAS    ---------   --------RLVPVGpGFRLLV  418
KSHV   376   RRAAMKGN[5]QRYAATDA---------RTLGSSTVSDMLEPT    ---------   --------KHVSLE-NFKITI  423
HHV6   394   --------   --------------SAVPPGVRPDFTFVAQLP    KRKELPNVP   GGIDFAEITSVRHG-AVILNA  440
HHV7   390   --------   --------------E-----INQQFSFEIPSY    QEKTLQFIP   GGNDFAEITSVTHG-ETTVNA  431

HCMV   561   FNTNRVINMKAALSSIAASGLgvRAPRL   PKNMTHSFVMYKHTFKEPACTVSTFVSNDAVYINSLNVNIRGSYPEFLY  637
MCMV   587   FNTNRVINLKATI---------vQTPRL[5]PRNMTHSFVMYKHTFKEPPYTVSTFVSNDAAHTNSLNVNIRGSYQEFLY  659
EBV    419   FNTNRVINTKLVCSEP-LVKM--RVCNV   -PRLINNFVARKYVVKETAFTVSLFFTDGVGANLAINVNISGTYLSFLL  491
KSHV   424   FNTNMVINTKISCHVPNTLQK--TILNI   -PRLTNNFVIRKYSVKEPSFTISVFFSDNMCQGTAININISGDMLHFLF  497
HHV6   441   FNTNKVMNLKATISKRANFVY----HRI   PKTMTHSFVMYKHTFKEPAFTVSTFVSNDDLDMSSLNINIRGPYCDFLY  513
HHV7   432   FNTNRVMNVKAALSGEIHCVL----HRI   PKSMTHSFVMYKRTFKEPSLTVSTFISNDDFTTSSLNINIRGPYCDFLY  504
```

```
HCMV   638   SLGVYRLHVNIDHFFLPAVVCNSNSSLDVHGLEDQAVIRSERSKVYWTTNFPCMISHTNNVNVGWFKAATAIVPRVSGAD   717
MCMV   660   ALSVYKLYVNIENFFLPASVCNSNSSLDVHGIEDQGVIRSERDKVYWTTNFPCMISNTDNINVGWFKAATAIIPKVSGVA   739
EBV    492   AMTSLRCFLPVEAIY-PAAVSNWNSTLDLHGLENQSLVRENRSGVFWTTNFPSVVSCQDGLNVSWFKAATATISRVHGRT   570
KSHV   498   AMGTLKCFLPIRHIF-PVSIANWNSTLDLHGLENQYMVRMGRKNVFWTTNFPSVVSSKDGLNVSWFKAATATISKVYGQP   576
HHV6   514   ALGVYKMHVSIQDLFLPAFVCNSNNSVDLQGLEDQDVVRNRKKKVYWITNFPCMISNANKVNVGWFKAGTGIIPRVSGKD   593
HHV7   505   ALGVYRLHVNIQDFFLPAFVCNSNNSMDLHGLENQGIVRKRKKKVYWITNFPCMISNSEKVNVGWFKAGTGIIPKVSGTD   584

HCMV   718   LEAILLKELSCIKNMRDVCIDYGLHRVFTQLELRNSYQIPFLAKQLVLFLRACLLKLHGREKRLQLDRLVFEAAQRGLFD   797
MCMV   740   LENVLLKELAYVTSIDQLCVDYTLHRVFTVLETRNCYQIPFLSKQFILFVRIMMLRICGLEHRLAVDRLIFRAIRQGVFD   819
EBV    571   LEQHLIREITPIVTHREAKISRIKNRLFTLLELRNRSQIQVLHKRFL----EGLLDCASLLRLDP--SCINRIASEGLFD   644
KSHV   577   LVEQIRHELAPILTDQHARIDGNKNRIFSLLEHRNRSQIQTLHKRFL----ECLVECCSFLRLDV--ACIRRAAARGLFD   650
HHV6   594   LQNVLLQELNNVREIPGLVFDMDLHQLLVLLEQRNLHQIPFLVKQFLIFLRLGLLMGYGHSRRNKVHDIMLHLISNGLFD   673
HHV7   585   LKNVLLKELISIGEIPNITFDMDLHALLTLLEKRNMHQVPFLIKQFFMFLRLGLLVGYGRKQERKVHHIMLFLIQKGFFD   664

HCMV   798   YSKNLTAHTKIKHTCALIGSRLANNVPKILARNKKVKLDHLGRNANVLTVCRHVEAHKIPRTRLkVLVEVLGVLQSISGT   877
MCMV   820   YHKNTVAHTKIKHTCALVGTRLANNVPKVLVKNKKIKLDYLGRNANLLTLCRHVDHACVDAHRLeALIGVLDCLEKLTSI   899
EBV    645   FSKRSIAHSKNRHECALLGHRHSANVTKLVVNERKTRLDILGRNANFLTRCKHQVNLRQSPIFL----TLLRHIRRRLGL   720
KSHV   651   FSKKIISHTKSKHECAVLGYKKCNLIPKIYARNKKTRLDELGRNANFISFVATTGH-RFAALKP----QIVRHAIRKLGL   725
HHV6   674   FNKNSVANTKIKHGCALVGTRLANNVPKIIARQKKMKLDHMGRNANSLAVLRFIVKSGEHKNKT-VFIKLLEYLAETSTA   752
HHV7   665   FSKNSVANSKIKHACALVGSRLANNVPKILSKQKKMKLDHLGRNANALTVLRFIVENGYYKRKT-IFRKLLKYLATTSFN   743

HCMV   878   PHTREVIHQTLFRLCSA---AAATSGLCSSPPpLCVSSSSS[26]   941
MCMV   900   DRTKEALTRARVRLCGGYRPEAATS----------------[ 2]   926
EBV    721   GRASVKREITLLLAHLRKKTAP-----------IHCRDAQV        750
KSHV   726   --------------HWRHRTAASNEQTPPADPrVRCVRPLV        752
HHV6   753   INTRNEVARLLQTLTTNMKT---------------------        772
HHV7   744   AHVQTESNRLLNLMHNDSKTNFSSLERLYTLR---------        775
```

9% identity overall

COBALT analysis of LTFc and TBP

```
HCMV   1     MAGA-[3]RLGC--DALIVVG--GSAMPrrvLHVPVHVRACNLTQELSTGEDARFCRPRPVNVERVRAVFAALYRACPIH   75
MCMV   1     MMSAS[5]SLSCldPALIVKS--PTA---rvKSAPVCVNSYNLTREISPFEDSRLSQAVTVDEEHISSIFRTLMAAGPDP   77
EBV    1     MTQGK[5]GLEG------------------------------FSSQLGLFYALACNRSPPALPEDATLLIKWLDTALGRE   52
KSHV   1     MA---   ALEG--PLLLPPSasLTTSP------QTTCDQATWESQLEIFCCLATNSHL-----QAELTLEGLDKMMQPE   61
HHV6   1     MQHTG   --NC--ETLIVNScfGSTCA---RSIPVFIDSCDLTAEVSRDEETRLARSVPVVLEKIESIIEKIFQTSGPN   70
HHV7   1     MIDSI   --SE--ETLIVKSytVNHCA---KNVPVFINSYDLTAEVAKNEDVRLARQVQISLEKIDEVIESIFSASGPS   70
TBP          -----   ------------------------------------------------------------------------

HCMV   76    VRTEPERVKLVLGRLLLGPVAVPCFCDG-EVEghgEHLVPTTQFCRGPLLYVHR-RCCCGSVtAGRALSYHVLENHVATH   153
MCMV   78    GATDEDKARVVLCRLLMGPVAVPCYCDEWDVD---DYLAKCAYRCSGPALYVHRSRCRCGA--EGGGTMFTLLHDHYTTH   152
EBV    53    ATFYACRA---MRRLLLGVIRIND-CQELPPG----LIILSPGTVPGPLGV----QSLEHTD-CEIWSSAHPDHAAHLP-   118
KSHV   62    PTFFACRA---IRRLLLG--------ERLHP------FIHQEGTLLGKVGRRYSGEGLIIDG-GGVFTRGQIDTDNYLPA   123
HHV6   71    IVHDKDRAKIALCRLLLGPVAVPCFCEEWDTN---DYLSKSGCKCLGPILYIHTSRCRCSDI---PVFKFSIMKDYYASH   144
HHV7   71    VENVKDQAKFALCRLLLGPVSIPCYCEEWDVN---FYLTKCSYNCEGPVLYIYKNASQCCE----STYRFSIMTNYHSTH   143
TBP          --------------------------------------------------------------------------------

HCMV   154   VLRGLLS   LTEWNRELPSLFCDCP[5]SGTEERYAMAC   LPRDLSLHLDDYPYLMVEIGRVLSVSEVDDYVTAV-S   228
MCMV   153   VFRGLLS   LSEWNVRLTDVFCACN   AFRSDRYVMAV   LPKHQSVFIEYYPYFLVCLARYLTVPEIDDCANSM-T   222
EBV    119   -------   --------VPRVITYT[5]ISTSSMFRLII[7]FERCFEQFCRVVPRRFLGTCKQNSAKMLAHLKQVTrI   186
KSHV   124   VGSWELT[8]FRELRSLYLPALLTCT[2]--YKAMFRIVC[7]FEQCFHAFLAVLPHSLQPTIYQNYFALLESLKHLS-F   208
HHV6   145   VFRGLLS   LKEWNTHLPNVLCTCE   LSMSDRYVATV   FPKQNSIYLEYYPYFLCYLCRYLTVIEIEQCTNDL-I   214
HHV7   144   IFRGLLS   LQEWNSHLSNILCTCS   NVTGDKYTATI   FPNNASIYLEYYPYFLCYLCKHLSIIDIEQCTNEL-I   213
TBP          -------   ----------------   -----------   -------------------------------------

HCMV   229   GYLGEAAAPRIQVHYKLLFGLNVRPQ--APCAldATRDFFLLELQKLWLGVEYHHEVTSEFFGRVLAQLHRDRARVMMAL   306
MCMV   223   AHLGPAIAARVGVHYKMLFGANARPPrvTEVArrANYDLFLLELQKLWLNVSYRNAVTRDFFETVFSAFHHETGKVMLAL   302
EBV    187   PPCPPFSGREARLKF---------------------HFFSWSTFMLSW----PNNATLREIRTRAATNLTHHPHLVDTLY   241
KSHV   209   SIMPP-ASPDAQLHF---------------------LKFNISSFMATW----GWHGELVSLRRAIAHNVERLPTVLKNLS   262
HHV6   215   SLLGPKVAQRVIIHFKLLFGFRHKPHm-GTVDswFWENFFMLELHKLWLTVVKHNRVTTDFFNVVYEKIQNYKQYAIKTL   293
HHV7   214   AFLGPKTSQRIIIHYKLLFGFRSKPMn-FTVS--LLEQVFTLEIQKLYYSVSKHNSTTADFFNVITAKFAEDKYFVLRTF   290
TBP          --------------------------------------------------------------------------------

HCMV   307   RLPEQTVCHLSTFVLSRFKRQV   LYFKLQVSYGKCRTGHAD[14]NLLCYRRLSVTFADTD--TVWRNLFYVYY[18]   405
MCMV   303   RSPGRQPLFPRWISMSRFKKQV   LYFELEVRCTKSRKDELK    NALIFRKTSVLFADHD--VIWRNLFYTYY[30]   399
EBV    242   HASPQTPFLTRSGALYRFVTCC[6]--ISIQQCKAGDRPGDLE[ 9]-----RPASFQFPSSPtgALLRCIVAAAL[ 3]   321
KSHV   263   KQSKHQDVKVNGRDLVGFQLAL[6]LHVKIQRKDPGPKPYRVV[ 5]-----CTYYLVYPGTP--AIYRLVMCMAV[ 3]   338
HHV6   294   RMSSKAVPAIQRFCLAKFKQQL   LYLNIKVTVKKNKRELCL    NGFVYGKTLYVVESSQ--LIFRNLLLLYY        360
```

```
HHV7  291  KLSAQITPGIQSFCSLKFKLQT   LYLNLKIM--KNTKLSIS    NSFYHGKTLYTLDEKQ--LVWRNLLLIYY      355
TBP        ----------------------   ------------------    ------------------------------

HCMV  406  GYGV[17]SANGHALSSTALPTTSAGH[11]RV   ------RRYVCIISRLMYARYG[5]HRQRRSET   -----GEE  490
MCMV  400  GGGV[26]TESGVAVSAPAAAATSAAK[ 9]TV[8]VSSTATQKYVKIVDRLALVRL-[4]RRLAAEAA[9]GAVAGGTS  517
EBV   322  ----    VSVGHQELSPLMSRSHGGQ    TD[7]-----ARRLVALLRREDGAPKD   PPLGPFG-   HPRGPGPA  381
KSHV  339  ----    IGHSCSGLHPCANFLGTHE    T-   -----PRLLAATLSRIRYAPKD   RRAAMKGN[5]QRYAATDA  396
HHV6  361  DYSL[ 7]-------------------    --   EENVLTTHYIRVISRLSFKQSR   --------   --------  393
HHV7  356  GYNL[ 8]-------------------    --   EESLLSMHYIRILERLSLKSFR   --------   --------  389
TBP   1    ----    -MDQNNSLPPYAQGLASPQ[11]PM[8]PQPIQNTNSLSILEEQQRQQQ[5]QQQQQQQQ[5]QQQQQQQQ  87

HCMV  491  EEEETLESGETDATPPFDFTGQQL[ 8]RRKHLAVQR[9]GGMEFAEVTGVSLD-RIAVNAFNTNRVINMKAALSSIAAS  579
MCMV  518  GVQTDVKADEPVAVPSFDFDPYRC[11]REGEGSVYG[5]GGREFSEMTAVSLN-RVAVNAFNTNRVINLKATI------  599
EBV   382  KSEDEESERRDAPPPPLDFSFQAS    ---------   ---------RLVPVGpGFRLLVFNTNRVINTKLVCSEP-LV  436
KSHV  397  ---------RTLGSSTVSDMLEPT    ---------   ---------KHVSLE-NFKITIFNTNMVINTKISCHVPNTL  442
HHV6  394  ------SAVPPGVRPDFTFVAQLP    KRKELPNVP   GGIDFAEITSVRHG-AVILNAFNTNKVMNLKATISKRANF  459
HHV7  390  ------E-----INQQFSFEIPSY    QEKTLQFIP   GGNDFAEITSVTHG-ETTVNAFNTNRVMNVKAALSGEIHC  450
TBP   88   QQQQQQQQAVAAAAVQQSTSQQAT[11]HSQTLTTAP[7]YPSPMTPMTPITPAtPASESSGIVPQLQNIVSTVNLGCKL  178

HCMV  580  GLgvRAPRL   PKNMTHSFVMYKHTFKEPACTVSTFVSNDAVYINSLNVNIRGSYPEFLYSLGVYRLHVNIDHFFLPAV  656
MCMV  600  ---vQTPRL[5]PRNMTHSFVMYKHTFKEPPYTVSTFVSNDAAHTNSLNVNIRGSYQEFLYALSVYKLYVNIENFFLPAS  678
EBV   437  KM--RVCNV   -PRLINNFVARKYVVKETAFTVSLFFTDGVGANLAINVNISGTYLSFLLAMTSLRCFLPVEAIY-PAA  509
KSHV  443  QK--TILNI   -PRLTNNFVIRKYSVKEPSFTISVFFSDNMCQGTAININISGDMLHFLFAMGTLKCFLPIRHIF-PVS  515
HHV6  460  VY----HRI   PKTMTHSFVMYKHTFKEPAFTVSTFVSNDDLDMSSLNINIRGPYCDFLYALGVYKMHVSIQDLFLPAF  532
HHV7  451  VL----HRI   PKSMTHSFVMYKRTFKEPSLTVSTFISNDDFTTSSLNINIRGPYCDFLYALGVYRLHVNIQDFFLPAF  523
TBP   179  DLktIALRA[2]AEYNPKRFAAVIMRIREPRTTALIFSSGKMVCTGAKSEE-QSRLAARKYARVVQKLGFPAK--FLDFK  254

HCMV  657  VCNSNSSLDVH-GLEDQAVIRSERS-KVYWTTNFPCMISHTNNVNVGWFKAATAIVPRVSGADLEAILLKELSCIKNMRD  734
MCMV  679  VCNSNSSLDVH-GIEDQGVIRSERD-KVYWTTNFPCMISNTDNINVGWFKAATAIIPKVSGVALENVLLKELAYVTSIDQ  756
EBV   510  VSNWNSTLDLH-GLENQSLVRENRS-GVFWTTNFPSVVSCQDGLNVSWFKAATATISRVHGRTLEQHLIREITPIVTHRE  587
KSHV  516  IANWNSTLDLH-GLENQYMVRMGRK-NVFWTTNFPSVVSSKDGLNVSWFKAATATISKVYGQPLVEQIRHELAPILTDQH  593
HHV6  533  VCNSNNSVDLQ-GLEDQDVVRNRKK-KVYWITNFPCMISNANKVNVGWFKAGTGIIPRVSGKDLQNVLLQELNNVREIPG  610
HHV7  524  VCNSNNSMDLH-GLENQGIVRKRKK-KVYWITNFPCMISNSEKVNVGWFKAGTGIIPKVSGTDLKNVLLKELISIGEIPN  601
TBP   255  IQNMVGSCDVKfPIRLEGLVLTHQQfSSYEPELFPGLIYRMIKPRIVLLIFVSGKVV-LTGAKVRAEIYEAFENIYPILK  333

HCMV  735  VCIDYGLHRVFTQLELRNSYQIPFLAKQLVLFLRACLLKLHGREKRLQLDRLVFEAAQRGLFDYSKNLTAHTKIKHTCAL  814
MCMV  757  LCVDYTLHRVFTVLETRNCYQIPFLSKQFILFVRIMMLRICGLEHRLAVDRLIFRAIRQGVFDYHKNTVAHTKIKHTCAL  836
EBV   588  AKISRIKNRLFTLLELRNRSQIQVLHKRFL----EGLLDCASLLRLDP--SCINRIASEGLFDFSKRSIAHSKNRHECAL  661
KSHV  594  ARIDGNKNRIFSLLEHRNRSQIQTLHKRFL----ECLVECCSFLRLDV--ACIRRAAARGLFDFSKKIISHTKSKHECAV  667
HHV6  611  LVFDMDLHQLLVLLEQRNLHQIPFLVKQFLIFLRLGLLMGYGHSRRNKVHDIMLHLISNGLFDFNKNSVANTKIKHGCAL  690
HHV7  602  ITFDMDLHALLTLLEKRNMHQVPFLIKQFFMFLRLGLLVGYGRKQERKVHHIMLFLIQKGFFDFSKNSVANSKIKHACAL  681
TBP   334  GFRKTT--------------------------------------------------------------------------  339

HCMV  815  IGSRLANNVPKILARNKKVKLDHLGRNANVLTVCRHVEAHKIPRTRLkVLVEVLGVLQSISGTPHTREVIHQTLFRLCSA  894
MCMV  837  VGTRLANNVPKVLVKNKKIKLDYLGRNANLLTLCRHVDHACVDAHRLeALIGVLDCLEKLTSIDRTKEALTRARVRLCGG  916
EBV   662  LGHRHSANVTKLVVNERKTRLDILGRNANFLTRCKHQVNLRQSPIFL----TLLRHIRRRLGLGRASVKREITLLLAHLR  737
KSHV  668  LGYKKCNLIPKIYARNKKTRLDELGRNANFISFVATTGH-RFAALKP----QIVRHAIRKLGL--------------HWR  728
HHV6  691  VGTRLANNVPKIIARQKKMKLDHMGRNANSLAVLRFIVKSGEHKNKT-VFIKLLEYLAETSTAINTRNEVARLLQTLTTN  769
HHV7  682  VGSRLANNVPKILSKQKKMKLDHLGRNANALTVLRFIVENGYYKRKT-IFRKLLKYLATTSFNAHVQTESNRLLNLMHND  760
TBP        --------------------------------------------------------------------------------

HCMV  895  ---AAATS[42]  941
MCMV  917  YRPEAATS[ 2]  926
EBV   738  KKTAP---[ 8]  750
KSHV  729  HRTAASNE[16]  752
HHV6  770  MKT-----      772
HHV7  761  SKTNFSSL[ 7]  775
TBP        --------
```

LTFd

>HCMV
MNSLLAELNRLGVAHATTEDVFIFVDRLFQHFSFLFQAEESGPRRLELVASVFEHLTVECVNDILDACSHPDVNVVETSNTCRPCPSPV
PSAPKTVSDAQTSCATPRAPVT

>MCMV
MVTFMVNTSEGDVMLLGVACIQVYKSAVPPLASAKHSSTPSSMNVILGELRKAGIEHETLEDVFRLAESIGEACDFFRQPGESRLRVLD
LAVSLFDHVAAECIGDVVSLGFVESQDGAPGGTTGHPRGATASKD

>EBV
MSAPGCSERQDKKRVTIGEREFGELLSWDPTDLPRTVARVYVAVGGLFEQEVSEVQRLENICTLLDLAGVECQTKAD

>KSHV
MGEPVDPGHVVNEKDFEECEQFFSQPLREQVVAGVRALDGLGLADSLCHKTERLCLLMDLVGTECFARVCRLDTGAK

>HHV6
MNSALNEIKDDFDNCETKNDLFKIIDKISKNCNFIVEQVESLPRRVDSAAILFDNLAVEIFNDVIYRQNGDGVPAKIRQGNGQNIDT

>HHV7
MNGVLNDIKTEFLCNTKTDLLTLIQKICLNCDFILEPVESFPKKTELVAVMYDTLAVEIFNDLLKYNEQKKDGLA

COBALT analysis of LTFd

```
HCMV  1        ----   ---MNSLLAELNRLGVAHATTEDVFIFVDRLFQHFSFLFQAEESGPRRLELVASVFEHLTVECVNDIL-  65
MCMV  1    [30]LASA[5]PSSMNVILGELRKAGIEHETLEDVFRLAESIGEACDFFRQPGESRLRVLDLAVSLFDHVAAECIGDVV-  107
EBV   1        MSAP[5]QDKKRVTIGEREFGELLSWDPTDLPRTVARVYVAVGGLFEQEVSEVQRLENICTLLDLAGVEC------  72
KSHV  1        MGEP   VDPGHV-VNEKDFEECEQFFSQPLREQVVAGVRALDGLGLAD-SLCHKTERLCLLMDLVGTECFARVCr  71
HHV6  1        ----   ---MNSALNEIKDDFDNCETKNDLFKIIDKISKNCNFIVEQVESLPRRVDSAAILFDNLAVEIFNDVI-  65
HHV7  1        ----   ---MNGVLNDIKTEFL-CNTKTDLLTLIQKICLNCDFILEPVESFPKKTELVAVMYDTLAVEIFNDLLk  65

HCMV  66   -DACSHPDVNVVETSNTCRPCPS[24]  111
MCMV  108  -------SLGFVESQDGAPGGTT[11]  134
EBV   73   -QTKAD-----------------      77
KSHV  72   lDTGAK-----------------      77
HHV6  66   -YRQNGDGVPAKIRQGNGQNIDT      87
HHV7  66   yNEQKKDGLA-------------      75
```

2% identity overall

LTFe

>HCMV
MCDASGACDMRHVQNAFTEEIQLHSLYACTRCFRTHLCDLGSGCALVSTLEGSVCVKTGLVYEALYPVARSHLLEPIEEAALDDVNIIS
AVLSGVYSYLMTHAGRYADVIQEVVERDRLKKQVEDSIYFTFNKVFRSMHNVNRISVPVISQLFIQLIIGIYSKQTKYDACVIKVSRKK
REDALLKQMRSEYGNAPVFGSGV

>MCMV
MFSHGRDPRTGKPISAAGARAHARGGGSGKCDGGGGCDMRNLCNPLTQELNLRNMYVCVRCHRTHLCDLRHDCVVVHTQDGSVCIKTGL
TYGSVFPGGCVSALEPVTEPHVDEINVVGVIMSYVYTYLTRNADHYADVIGSVIEGGWFNKPTENAIYFTFNRVFKQHNALQKVPISVI
GQLFVQLVIGVHARVTKYDSTVIKVSRRKREDGLLKRMRFEYGNAPSFRTGR

>EBV
MSDQGRLSLPRGEGGTDEPNPRHLCSYSKLEFHLPLPESMASVFACWGCGEYHVCDGSSECTLIETHEGVVCALTGNYMGPHFQPALRP
WTEIRQDTQDQRDKWEPEQVQGLVKTVVNHLYHYFLNENVISGVSEALFDQEGALRPHIPALVSFVFPCCLMLFRGASSEKVVDVVLSL
YIHVIISIYSQKTVYGALLFKSTRNKRYDAVAKRMRELWMSTLTTKC

>KSHV
MKSVASPLCQFHGVFCLYQCRQCLAYHVCDGGAECVLLHTPESVICELTGNCMLGNIQEGQFLGPVPYRTLDNQVDRDAYHGMLACLKR
DIVRYLQTWPDTTVIVQEIALGDGVTDTISAIIDETFGECLPVLGEAQGGYALVCSMYLHVIVSIYSTKTVYNSMLFKCTKNKKYDCIA
KRVRTKWMRMLSTKDT

>HHV6
MEMAFPRKYDRVTGRILTHKNNQMCTTECSQMYNLHNPITFELGLGNVFVCMRCLTVHHCDMQTDCTIVNTHEGYVCAKTGLFYSGWMP
TYADCFLEPICEPNIETVNVVVVLLSYVYSFLMENKERYAAIIDSIIKDGKFIKNVEDAVFYTFNAVFTNSTFNKIPLTTISRLFVQLI
IGGHAKGTIYDSNVIRVSRRKREDSLLKKMRLEYGNALIL

>HHV7
MNKKKMDLPKCQSITVACEGECSQMYNLHNPLTFEMGLGNIFICVRCFKIHFCNMLEDCNLINTHEGCVCSKTGLFYNGWMPAYSHTCM
EPTEEPNMETVNVVVVLLSYVYSFLIQNKARYSNIIRDIIKDGKFIEQVENAVFCTFNKVFKNSTLNKLPLTTVSQLFVQLIIGGHAEG
TIYDNNVIRVSRRKREDNILKKMRIEYGNALAL

COBALT analysis of LTFe

(Bold residues are involved in Zn binding)

```
HCMV   1        ----------------MCDASGA------CDMRHVQNAFTEEIQLHSLYACTRCFRTHLCDLGSGCALVSTLEGSV   54
MCMV   1    [13]ISAAgaRAHARGGGSGKCDGGGG------CDMRNLCNPLTQELNLRNMYVCVRCHRTHLCDLRHDCVVVHTQDGSV   83
EBV    1        MSDQ--GRLSLPRGEGGTDEPNP---RHLCSYSKLEFHLPLPESMASVFACWGCGEYHVCDGSSECTLIETHEGVV   71
KSHV   1        --------------------------------MKSVASPLCQFHGVFCLYQCRQCLAYHVCDGGAECVLLHTPESVI   45
HHV6   1        MEMAfpRKYDRVTGRILTHKNNQmctTECSQMYNLHNPITFELGLGNVFVCMRCLTVHHCDMQTDCTIVNTHEGYV   76
HHV7   1        MNK---KKMDLPKCQSITVA----ceGECSQMYNLHNPLTFEMGLGNIFICVRCFKIHFCNMLEDCNLINTHEGCV   69

HCMV   55   CVKTGLVYEALYPVARSHLLEPIEEAALDDVN    IISAVLSGVYSYLMTHAGRYADVIQEVVERDR-LKKQVEDSIYF   129
MCMV   84   CIKTGLTYGSVFPGGCVSALEPVTEPHVDEIN    VVGVIMSYVYTYLTRNADHYADVIGSVIEGGW-FNKPTENAIYF   158
EBV    72   CALTGNYMGP--HFQ--PALRPWTEIRQDTQD[12]LVKTVVNHLYHYF-LNENVISGVSEALFDQEGaLRPHIPALVSF   154
KSHV   46   CELTGNCMLG--NIQEGQFLGPVPYRTLDNQV[ 7]MLACLKRDIVRYL-QTWPDTTVIVQEIALGDG-VTDTISAIIDE   124
HHV6   77   CAKTGLFYSGWMPTYADCFLEPICEPNIETVN    VVVVLLSYVYSFLMENKERYAAIIDSIIKDGK-FIKNVEDAVFY   151
HHV7   70   CSKTGLFYNGWMPAYSHTCMEPTEEPNMETVN    VVVVLLSYVYSFLIQNKARYSNIIRDIIKDGK-FIEQVENAVFC   144

HCMV   130  TFN---KVFRSMHNvNRISVPVISQLFIQLIIGIYSKQTKYDACVIKVSRKKREDALLKQMRSEYGNAPVFGSGv   201
MCMV   159  TFNRVFKQHNALQK---VPISVIGQLFVQLVIGVHARVTKYDSTVIKVSRRKREDGLLKRMRFEYGNAPSFRTGr   230
EBV    155  VFPCCLMLFRGASS-EK-VVDVVLSLYIHVIISIYSQKTVYGALLFKSTRNKRYDAVAKRMRELWMSTLTTKC--   225
KSHV   125  TFGECLPVLGEAQG----GYALVCSMYLHVIVSIYSTKTVYNSMLFKCTKNKKYDCIAKRVRTKWMRMLSTKDT-   194
HHV6   152  TFN---AVFTNSTF-NKIPLTTISRLFVQLIIGGHAKGTIYDSNVIRVSRRKREDSLLKKMRLEYGNALIL----   218
HHV7   145  TFN---KVFKNSTL-NKLPLTTVSQLFVQLIIGGHAEGTIYDNNVIRVSRRKREDNILKKMRIEYGNALAL----   211
```

8% identity overall

LTFf

>HCMV
MMAAAVVRAEVRRQRREERKKMASARTTEDPPENHVVADVACGTGAVTRSSSSSLVVSSSSASGSDESSSASPLSFPVSSPSTAVRSPG
SAGVSTSLCSVERMVELSAQSPAADFSVSEAWRFEEAVNMALVACEAVSPYDRFRLIETPDENFLLVTNVIPRESAEVPVLDSSSSGGD
SGPEDKKKNVGNKTAGEKNGGGSRAKRRRRRRAPKNDAATPSFLRRHDVLERFAAAAEPLPSLCVHDYALRNADRVTYDGELIYGSYLL
YRKAHVELSLSSNKVQHVEAVLRQVYTPGLLDHHNVCDVEALLWLLYCGPRSFCARDTCFGREKNGCPFPALLPKLFYEPVRDYMTYMN
LAELYVFVWYRGYEFPAPTPQATTAGGGGGGGSGGGGGAGACAVETSASAGRVDDAGDEVHLPLKPVSLDRLREVLQAVRGRFSGREVP
AWPASSRTCLLCALYSQNRLCLDLARDEARTVSYSPIVIQDCAAAVTDVTLSHILPGQSTVSLFPVYHVGKLLDALSLNDAGLITLNL

>MCMV
MATAAGGVYAGGEDQQQQRPRATDVSTALCDVEALAAVEEGRVSEADVNRYREAVDAALIACEASSPRDRFRLIETAGGNFLLVTNALP
KDRTEQQPPCVLEGSGRASSNNNYEGIGTPSAGSGNAFDGLLALERGTSGGGLTATVPSAPGYVAKSVNTLSYDGRLLSNSYVLYTKEQ
LRKSLSPDKRAIVERILRFVDTPGILDHNNVQDVEAVLWLLFCGPQSVCQNPTCFGRDRECEVSYPVLLPPVFYDPITDYSAYINLAEL
YVYVWYRNYDFDSEPTRCYELGTVAMDRVKKTLQSVRQRFSDRSVPVWPVSSRTCVFCALYNQNRVCLDLAKSDVDVTSYSPIIIKDCR
DAATNVTLSHVLPGQRVASLFPVYDIGTLLRALCDSNDGEERRKRMRETIDSALSTTDDAV

>EBV
MFNAVKADMPDDPMLARRYGQCLELALEACQDTPEQFKLVETPLKSFLLVSNILPQDNRPWHEARSSGRVAEDDYDFSSLALELLPLNP
RLPEEWQFGGQGWSSRMEPSQPEMGMGLCFEVFDGDLMRIALAWNKDEVIGQALQILAHSHTWTSLVPEDPLPWMWALFYGPRSHCEER
HCVYAAARGKRGPILLPTAVYTPCANIEAFLAHLTRCVYALYLDVRDWKGEDIAPPFDVSRLNKMAKQLCLLPQEPFCITRVCLLCLLH
KQNLNAQYKRPVDTYDPCLILTGEAERYMVDAVGNYREASTGTTVLYPTYDLGSIVADMVTYEDE

>KSHV
MFALSSLVSEGDPEVTSRYVKGVQLALDLSENTPGQFKLIETPLNSFLLVSNVMPEVQPICSGRPALRPDFSNLHLPRLEKLQRVLGQG
FGAAGEEIALDPSHVETHEKGQVFYNHYATEEWTWALTLNKDALLREAVDGLCDPGTWKGLLPDDPLPLLWLLFNGPASFCRADCCLYK
QHCGYPGPVLLPGHMYAPKRDLLSFVNHALKYTKFLYGDFSGTWAAACRPPFATSRIQRVVSQMKIIDASDTYISHTCLLCHIYQQNSI
IAGQGTHVGGILLLSGKGTQYITGNVQTQRCPTTGDYLIIPSYDIPAIITMIKENGLNQL

>HHV6
MEPDLTLAAVYQAAANLTEQDKEIFSEAVKTAFSVCSSAAPSARLRMIETPTQNFMFVTSVIPSGVPSGEKKTKLNIDAALDNLALSFA
NKKSKKMARTYLLQNVSRTQDQQVAISGTYILYTKKHIETSLMLDKTKLVKQILEYAETPNLLGYTDVRDLECLLWLVFCGPKSFCQSD
SCFGYSKTGYNAAFPNLLPPYLYECGQNNGLFFGIVQAYVFSWYSDFDFSALEISERARRRIRSLLYDLKQKFSEQEISVLPVASQMCI
FCALYKQNKLSLEYVSGDLKTSVFSPIIIKDCLCVQTTISTTQMLPGTKSSAIFPVYDLRKLLSALVISEGSVRFDI

>HHV7
MDTDIALAAIYKETTKLNEKDAKIFSEAVQTALTVCKATAPNTRLKLVETPTNNFLLVTNVVPSETSKATTEANLNIDAALEKLASSFN
TAVPVKSSKKYLLQNVRKMTSENIALTGSYIIYTKKHIEVAFLLDKSDFVQDILRYAETPSLLGHTDVRDLECLLWLAFCGPMSYCQAD
NCFGLNKAGYNAPFPILFPPCMYERNMNLSVFFGLLQIYVFSLYRDFSVENSNLQQGIKKRIKLVLSDLRAKERICEEEIGNFPLAAQI
CLFCALYRQNRLCMEYAANNLSMSVFSPIILKDCTFMQTTVTITQILPGSKEAIIFPVYDIGKLLSALVFSENGVLLKL

COBALT analysis of LTFf

(Bold residues are involved in Zn binding)

```
HCMV   1     [96]LCSVERMVELSAQSPAADFSVSEawRFEEAVNMALVACEAVSPYDRFRLIETPDENFLLVTNVIPreSAEVPVLDS  172
MCMV   1     [28]LCDVEALAAVEE--GRVSEADVN--RYREAVDAALIACEASSPRDRFRLIETAGGNFLLVTNALPkdRTEQQPPCV  100
EBV    1         ------MFNAVKADMPDDPMLAR--RYGQCLELALEACQDTP--EQFKLVETPLKSFLLVSNILP--QDNRPWHEA  64
KSHV   1         ------MFALSSLVSEGDPEVTS--RYVKGVQLALDLSENTP--GQFKLIETPLNSFLLVSNVMP--EVQPIC---  61
HHV6   1         MEPDLTLAAVYQAAANLTEQDKE--IFSEAVKTAFSVCSSAAPSARLRMIETPTQNFMFVTSVIP-----------  63
HHV7   1         MDTDIALAAIYKETTKLNEKDAK--IFSEAVQTALTVCKATAPNTRLKLVETPTNNFLLVTNVVP-----------  63

HCMV   173   SSSGGDSGPEDKKKNVGN[36]VLERFAAA-AEPLPSLCVHDYALRNADRVTYDG    ELIYGSYLLYRKAHVELSLSS  279
MCMV   101   LEGSGRASSNNNYEGIGT[13]ALERGTSGGGLTATVPSAPGYVAKSVNTLSYDG    RLLSNSYVLYTKEQLRKSLSP  185
EBV    65    RSSGRVAEDDYDFSSL--    ------------------ALELLPLNPRLPEEW[19]--MGLCFEVFDGDLMRIALAW  133
KSHV   62    -SGRPALRP--DFSNL--    ------------------HLPRLEKLQRVLGQG[20]GQVFYNHYATEEWTW--ALTL  128
HHV6   64    --SGVPSGEKKTKLNIDA    ALDNLALSFANKKSKKMARTYLLQNVSRTQDQQ    VAISGTYILYTKKHIETSLML  133
HHV7   64    --SETSKATTEANLNIDA    ALEKLASSFNTAVPVKSSKKYLLQNVRKMTSEN    IALTGSYIIYTKKHIEVAFLL  133

HCMV   280   NKVQHVEAVLRQVYTPGLLDHHNVCDVEALLWLLYCGPRSFCARDTCFGREKNG--CPFPALLPKLFYEPVRDYMTYMNL  357
MCMV   186   DKRAIVERILRFVDTPGILDHNNVQDVEAVLWLLFCGPQSVCQNPTCFGRDREC-EVSYPVLLPPVFYDPITDYSAYINL  264
EBV    134   NKDEVIGQALQILAHSHTWTSLVPEDPLPWMWALFYGPRSHCEERHCVYAAARG--KRGPILLPTAVYTPCANIEAFLAH  211
KSHV   129   NKDALLREAVDGLCDPGTWKGLLPDDPLPLLWLLFNGPASFCRADCCLYKQHCG--YPGPVLLPGHMYAPKRDLLSFVNH  206
HHV6   134   DKTKLVKQILEYAETPNLLGYTDVRDLECLLWLVFCGPKSFCQSDSCFGYSKTGyNAAFPNLLPPYLYECGQNNGLFFGI  213
HHV7   134   DKSDFVQDILRYAETPSLLGHTDVRDLECLLWLAFCGPMSYCQADNCFGLNKAGyNAPFPILFPPCMYERNMNLSVFFGL  213

HCMV   358   AELYVFVWYRGYE[39]DDAGDEVHLplkPVSLDRLREVLQAVRGRFSGREVPAWPAS    SRTCLLCALYSQNRLCLDL  469
MCMV   265   AELYVYVWYRNYD[ 1]DSEPTRCYE-lgTVAMDRVKKTLQSVRQRFSDRSVPVWPVS    SRTCVFCALYNQNRVCLDL  337
EBV    212   LTRCVYALYLDVR    DWKGEDIAP---PFDVSRLNKMA---------KQLCLLPQE[4]TRVCLLCLLHKQNLNAQYK  276
KSHV   207   ALKYTKFLYGDFS    GTWAAACRP---PFATSRIQRVV---------SQMKIIDAS[4]SHTCLLCHIYQQNSIIAGQ  271
HHV6   214   VQAYVFSWYSDFD    FSALEISER-----ARRRIRSLLYDLKQKFSEQEISVLPVA    SQMCIFCALYKQNKLSLEY  281
HHV7   214   LQIYVFSLYRDFS[ 7]QGIKKRIKL---VLSDLRAKERI-------CEEEIGNFPLA    AQICLFCALYRQNRLCMEY  283

HCMV   470   ARDEARTVSYSPIVIQDCAAAVTDVTLSHILPGQSTVSLFPVYHVGKLLDALSLNDAGLITLN[ 1]  533
MCMV   338   AKSDVDVTSYSPIIIKDCRDAATNVTLSHVLPGQRVASLFPVYDIGTLLRALCDSNDGEERRK[17]  417
EBV    277   RPVD--TYDPCLILTGEAERYMVDAVGNYREASTGTTVLYPTYDLGSIVADMVTYEDE-----      332
KSHV   272   --GT--HVGGILLLSGKGTQYITGNVQTQRCPTTGDYLIIPSYDIPAIITMIKENGLNQL---      327
HHV6   282   VSGDLKTSVFSPIIIKDCLCVQTTISTTQMLPGTKSSAIFPVYDLRKLLSALVISEGSVRFDI      344
HHV7   284   AANNLSMSVFSPIILKDCTFMQTTVTITQILPGSKEAIIFPVYDIGKLLSALVFSENGVLLKL      346
```

5% identity overall
