# Supplementary material for: Structure Prediction of Complexes Controlling Beta- and Gamma-Herpesvirus Late Transcription Using AlphaFold 3
Source: Viruses. 2025 May 29;17(6):779. doi: 10.3390/v17060779 (PMC12197705; doi:10.3390/v17060779)
Supplement: Supplementary file 1 [file viruses-17-00779-s001.zip › Supplementary Figures.pdf]

Structure Prediction of Complexes Controlling Beta- and Gamma-herpesvirus Late Transcription using AlphaFold 3

David H. Price, Department of Biochemistry and Molecular Biology, University of Iowa, Iowa City, IA 52240

Supplemental Figures 1-4

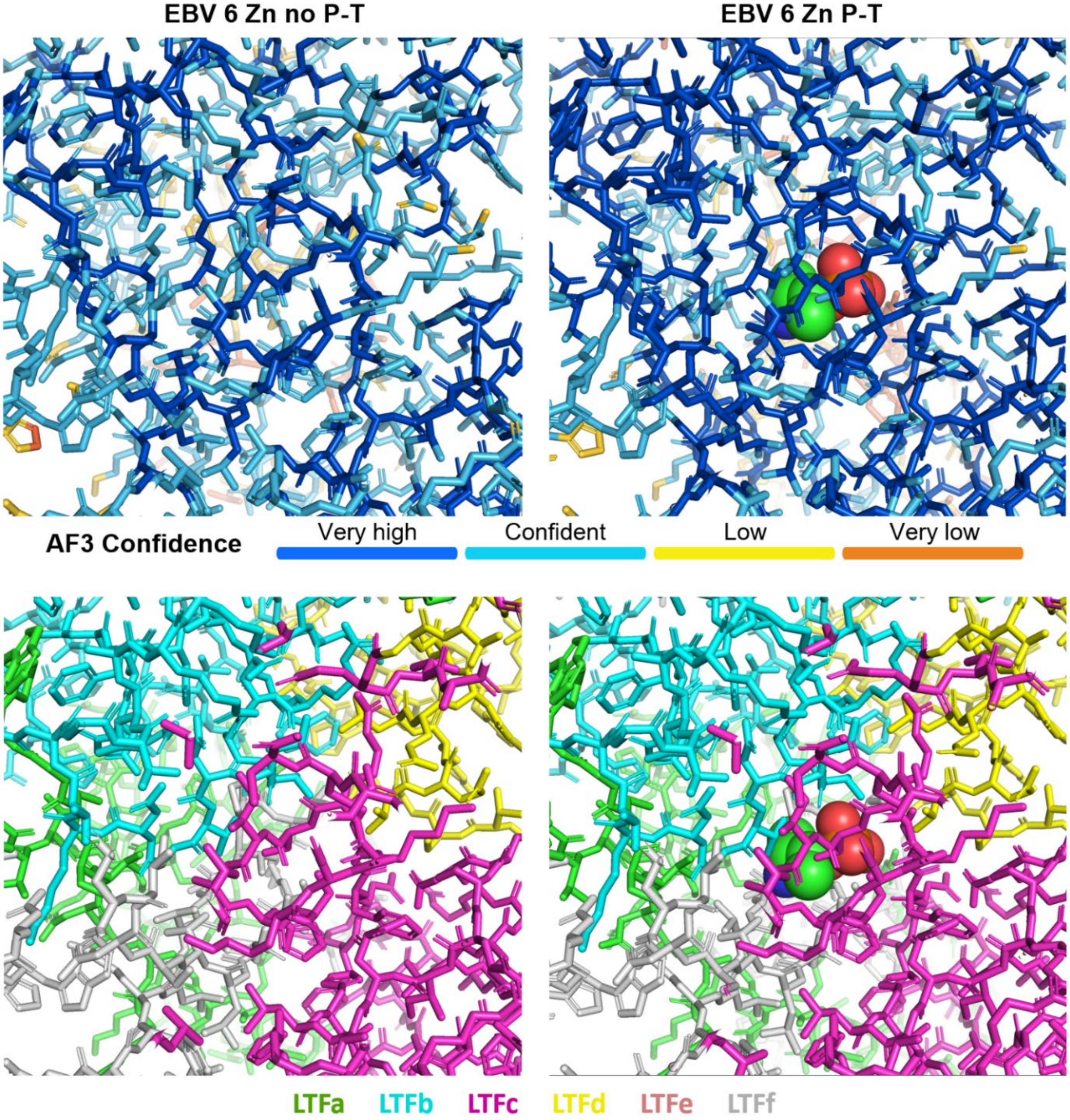

Supplementary Figure S1. Effect of LTFf phosphorylation on the EBV LTF complex. The EBV LTF complex was created with and without threonine phosphorylation (both in the presence of 6 zincs) and colored either by AlphaFold 3 confidence (top) or LTF chains (bottom).

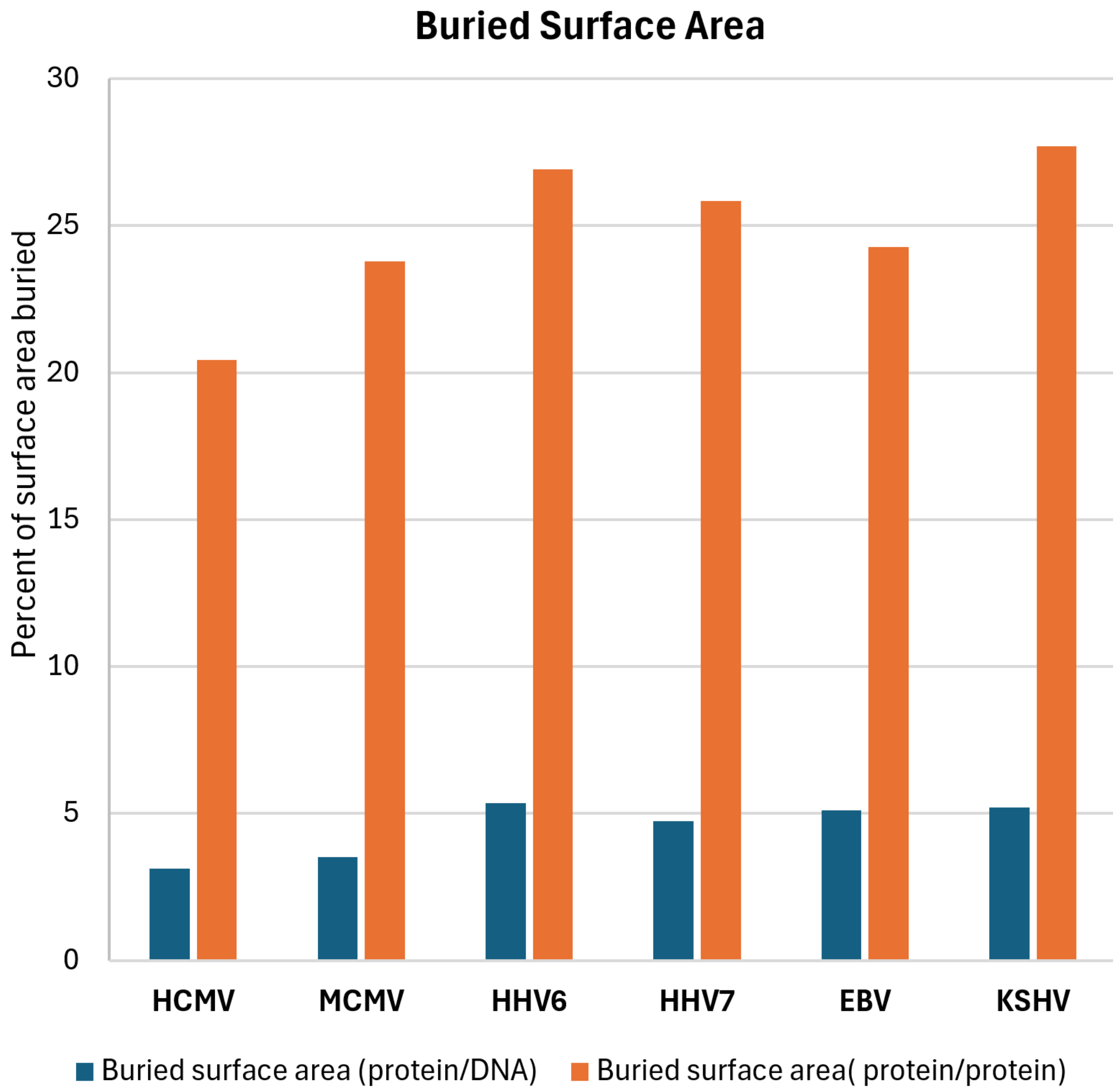

Supplementary Figure S2. Buried surface areas of LTF complexes. LTF complexes were generated by AlphaFold 3 for the indicated viruses with the 3 bp DNA fragment containing the TATTA element, LTFf threonine phosphorylation, and the optimum number of zinc atoms. The percentage of buried surface area between proteins and DNA and between proteins was calculated as described in Material and Methods.

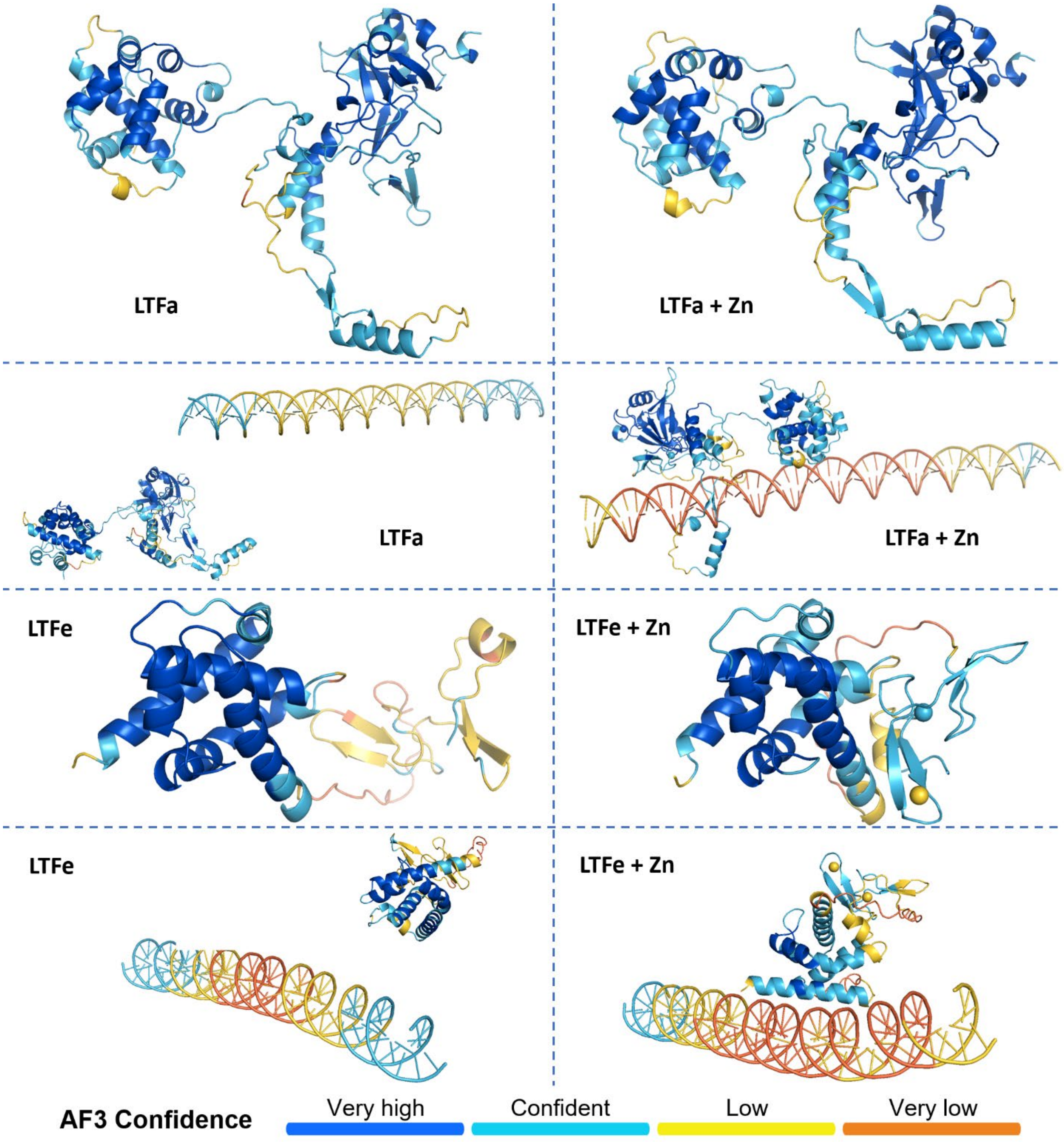

Supplementary Figure S3. Structural predictions of KSHV LTFa and LTFe with and without Zn and promoter DNA. Structures were colored with AlphaFold 3 confidence colors.

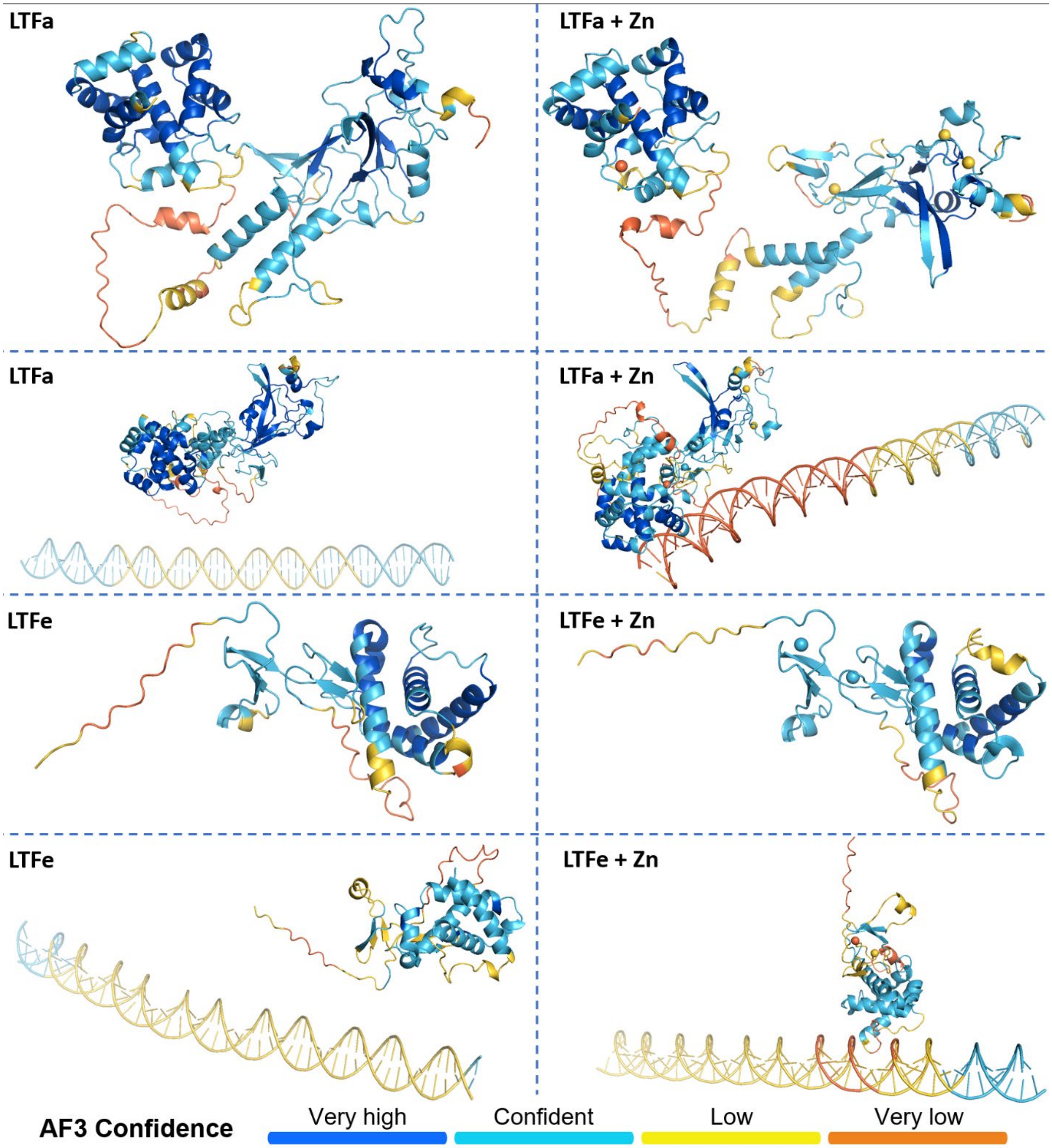

Supplementary Figure S4. Structural predictions of HHV7 LTFa and LTFe with and without Zn and promoter DNA. Structures were colored with AlphaFold 3 confidence colors.
